# Neural and Behavioral Correlates of Individual Variability in Rat Helping Behavior: A Role for Social Affiliation and Oxytocin Receptors

**DOI:** 10.1523/JNEUROSCI.0845-24.2025

**Published:** 2025-04-28

**Authors:** Reut Hazani, Jocelyn M. Breton, Estherina Trachtenberg, Keren Ruzal, Bar Shvalbo, Ben Kantor, Adva Maman, Einat Bigelman, Steve Cole, Aron Weller, Inbal Ben-Ami Bartal

**Affiliations:** ^1^Psychology Department, Bar-Ilan University, Ramat Gan 5290002, Israel; ^2^Gonda Brain Research Center, Bar-Ilan University, Ramat Gan 5290002, Israel; ^3^Geha Mental Health Center, Petah Tikva 4917002, Israel; ^4^Department of Psychology, Northeastern University, Boston, Massachusetts 02115; ^5^School of Psychological Sciences, Tel Aviv University, Tel Aviv 6997801, Israel; ^6^Sagol School of Neuroscience, Tel Aviv University, Tel Aviv 6997801, Israel; ^7^University of California, Los Angeles (UCLA) School of Medicine, Los Angeles, California 90095

**Keywords:** neural network, nucleus accumbens, oxytocin receptor, prosocial, social affiliation

## Abstract

A prosocial response to others in distress is increasingly recognized as a natural behavior for many social species. While prosocial behavior is more frequently observed toward familiar conspecifics, even within the same social context, some individuals are more prone to help than others. In a rat helping behavior test where animals can release a distressed conspecific trapped inside a restrainer, most rats are motivated and consistently release the trapped rat (“openers”), yet ∼30% do not open the restrainer (“nonopeners”). To characterize the difference between these populations, behavioral and neural markers were compared between opener and nonopener rats in males and females. Openers showed significantly more social affiliative behavior both before and after door opening compared with nonopeners. Oxytocin receptor mRNA levels were higher in the nucleus accumbens (NAc), but not the anterior insula, of openers. Several transcription control pathways were significantly upregulated in openers’ NAc. Chemogenetically inhibiting paraventricular oxytocin neurons did not significantly impair helping but reduced sociality measures, indicating that helping does not rely solely on oxytocin signaling. Analysis of brain-wide neural activity based on the immediate-early gene c-Fos in males revealed increased activity in openers in prosocial brain regions compared with nonopeners. These include regions associated with empathy in humans (insula, somatosensory, cingulate, and frontal cortices) and motivation and reward regions such as the NAc. These findings indicate that prosocial behavior may be predicted by affiliative behavior and activity in the prosocial neural network and provide targets for the investigation of causal mechanisms underlying prosocial behavior.

## Significance Statement

Prosocial behavior is observed in many social species, including rodents, yet the determinants underlying why some animals help and others do not are poorly understood. Here, we show behavioral and neural differences between prosocial and nonprosocial pairs in a rat helping behavior test, with increased social interaction and nucleus accumbens oxytocin receptor gene expression in animals that helped.

## Introduction

The motivation to help distressed others has been increasingly demonstrated across social species (Rault, 2019; Wu and Hong, 2022), extending beyond parental care and bonded pairs to conspecifics of the same social group (Decety et al., 2016). The neural mechanisms underlying a prosocial response involve processing cues of distress in others, which may elicit empathic arousal in the observer and motivate acts to improve the others’ well-being, such as consolation or targeted helping. The response to a distressed conspecific is critically different than social interaction in neutral contexts and, in many species (rodents, primates, elephants, corvids), elicits approach and consolatory touch (de Waal and Preston, 2017), which is considered effective in reducing distress. In recent years, more sophisticated prosocial acts have been demonstrated, which require actions congruent with the conspecific's goals such as food sharing (Hernandez-Lallement et al., 2014; Marquez et al., 2015; Brucks and von Bayern, 2020; Nafcha et al., 2023) and rescue behaviors (Moscovice et al., 2004; Ben-Ami Bartal et al., 2011; Sato et al., 2015; Zhang et al., 2024). These behaviors are preferentially demonstrated for affiliated others, either on the individual or group level. However, in these experiments, some proportion of subjects fail to show prosocial behavior. These individual differences could be explained by reduced sociability or prosocial motivation, failure to learn the task, high levels of personal distress, low trait empathy, or specific social dynamics. In this study, we aimed to understand determinants of prosocial behavior by outlining the behavioral and neural differences between prosocial and nonprosocial pairs in a rat helping behavior test (HBT), whereby rats can help free a trapped conspecific by opening a restrainer door. In the HBT, ∼50–70% of rats typically release a trapped cagemate, and once they learn to open the restrainer door, they tend to help quickly and consistently in the following sessions (Ben-Ami Bartal et al., 2011; Breton et al., 2022). Helping depends on the transfer of distress between the free and trapped rats (Ben-Ami Bartal et al., 2016), and moreover, rats selectively help affiliated others, not releasing trapped strangers of an unfamiliar strain (Ben-Ami Bartal et al., 2014). The role of distress in motivating helping, combined with the social selectivity of the behavior, is suggestive of empathic processes, where the affective state of one individual induces a congruent state in the observer, coupled with the prosocial motivation to act for their well-being.

The neural network recruited during the HBT involves regions of the human empathy network, including the anterior cingulate cortex (ACC) and anterior insula (AI), regions also found in rodent studies of empathy (Jeon et al., 2010; Carrillo et al., 2019; Smith et al., 2021). In addition, activity in the reward network correlates with helping (Ben-Ami Bartal et al., 2021). The nucleus accumbens (NAc) is a main hub of this “prosocial brain network,” with increased NAc activity observed in the presence of trapped ingroup members (cagemates or strangers of the same strain) compared with outgroup members (strangers of an unfamiliar strain) who weren't helped (Ben-Ami Bartal et al., 2021). Each of these regions contains a high density of oxytocin (OXT) receptors (Williams et al., 2020; Almeida et al., 2022), providing a potential link between prosocial behaviors and social affiliation networks, yet whether social affiliation or oxytocinergic signaling can distinguish individual differences in prosociality has yet to be explored.

Here, the brain-wide response during the HBT was compared between helper rats who consistently released trapped cagemates (“openers”) and rats who did not release cagemates (“nonopeners”) within the same social condition. To investigate whether oxytocin (OXT) signaling could account for differences in prosociality, OXT receptor mRNA levels were measured in the NAc and AI via RNA-seq and qPCR, and a chemogenetic experiment inhibiting paraventricular nucleus (PVN) OXT neurons was performed. Behavioral metrics of social affiliation were measured throughout to assess whether social relationship strength was predictive of future helping or associated with neural activity markers.

## Materials and Methods

### Experimental design

#### Animals

Experiment 1 included a total of 32 male and 32 female adult Wistar rats (Envigo RMS) and was performed at Bar-Ilan University. The experiment was performed in three batches to ensure consistency with replication. All rats arrived at the animal facility on postnatal day 52 and acclimated to the facility and their cagemate for 2.5 weeks before behavioral testing began. They were housed in same-sex pairs and received water and food *ad libitum*. The room had a controlled temperature of 22 ± 2°C and a 12 h light/dark cycle (lights on at 07:00 A.M.). At the beginning of the test phase, the rats’ weights were 192–245 g for females and 310–423 for males, and weights were recorded once a week throughout the experiment. The study protocol conformed to Society for Neuroscience guidelines and was approved by the Institutional Animal Use and Care Committee at Bar-Ilan University.

Experiment 2 included a total of 34 adult male Sprague Dawley (SD) rats (17 free rats, 17 trapped rats; Charles River Laboratories) and was performed at Tel Aviv University. All rats were approximately 3 months of age at the start of testing. Animals were housed in same-sex pairs, received food and water *ad libitum*, and were held in temperature and humidity-controlled housing rooms on a 12 h light/dark cycle. This experiment was performed in accordance with protocols approved by the Institutional Animal Use and Care Committee at Tel Aviv University.

Experiment 3 included 21 adult male Sprague Dawley (SD) rats (Charles River Laboratories). Data collection on 13 animals was performed at the University of California, Berkeley. Data from these animals have been previously published using different analyses, and full details can be found in prior work (Ben-Ami Bartal et al., 2021). This experiment was performed in accordance with protocols approved by the Institutional Animal Care and Use Committee at the University of California, Berkeley. A separate group of eight control animals was tested at Tel Aviv University; procedures were carried out in accordance with protocols approved by the Institutional Animal Use and Care Committee at Tel Aviv University.

In all experiments, every effort was made to minimize animal suffering and to reduce the number of animals used.

#### Stereotactic surgeries

In Experiment 2, 12 male SD rats (age, 3 months) underwent stereotactic injection of a viral vector containing inhibitory designer receptors exclusively activated by designer drugs (DREADDs) under an oxytocin promotor (AAV1/2-OTp-hM4D(Gi)-mCherry, a gift from Prof. Valery Grinevich's laboratory), targeting the periventricular nucleus (PVN; A/P −1.8, M/L ±0.35, D/V −8.0). This virus has been validated previously, with activation of the DREADD receptor leading to physiological downregulation of OXT neurons in the PVN (Eliava et al., 2016; Ferretti et al., 2019). Five additional rats were injected with a control virus lacking the chemogenetic receptor (AAV1/2-OTp-mCherry). Rats were first anesthetized with isoflurane (induction, 3–5%; maintenance, 1–3%) and mounted onto a stereotaxic frame. The skull was exposed following subcutaneous (s.c.) injection of lidocaine (5 mg/kg, 2%), and two small holes were made above the determined stereotactic coordinates. A Hamilton syringe containing the virus was used to inject 0.4 µl in each hemisphere. Following surgery, the rats were given subcutaneous injections of pain relievers (1 mg/kg of meloxicam 0.5%, 0.05 mg/kg of buprenorphine 0.3 mg/ml) and saline (10 ml/kg) to ensure hydration. The rats were allowed 2 weeks to recover and then started the habituation and the HBT protocol (described below), allowing 3 weeks in total for viral expression.

#### Behavioral testing

All procedures were undertaken during daylight hours. Throughout testing days, the pairs were tested in a counterbalanced order to ensure that the testing order did not bias their behavior. The testing arena was made of white Polygal (width, depth, height: 50 cm × 50 cm × 70 cm). All behavioral apparatuses were sanitized with a 70% ethanol solution at the end of each test period to remove odor residue. Tests were filmed using a video camera connected to EthoVision XT 15 software (Noldus). Manual behavior analysis was coded using the Solomon Coder software (Péter, 2011) for Experiment 1 and BORIS software (https://www.boris.unito.it) for Experiments 2 and 3.

##### Habituation

**Figure 1.**
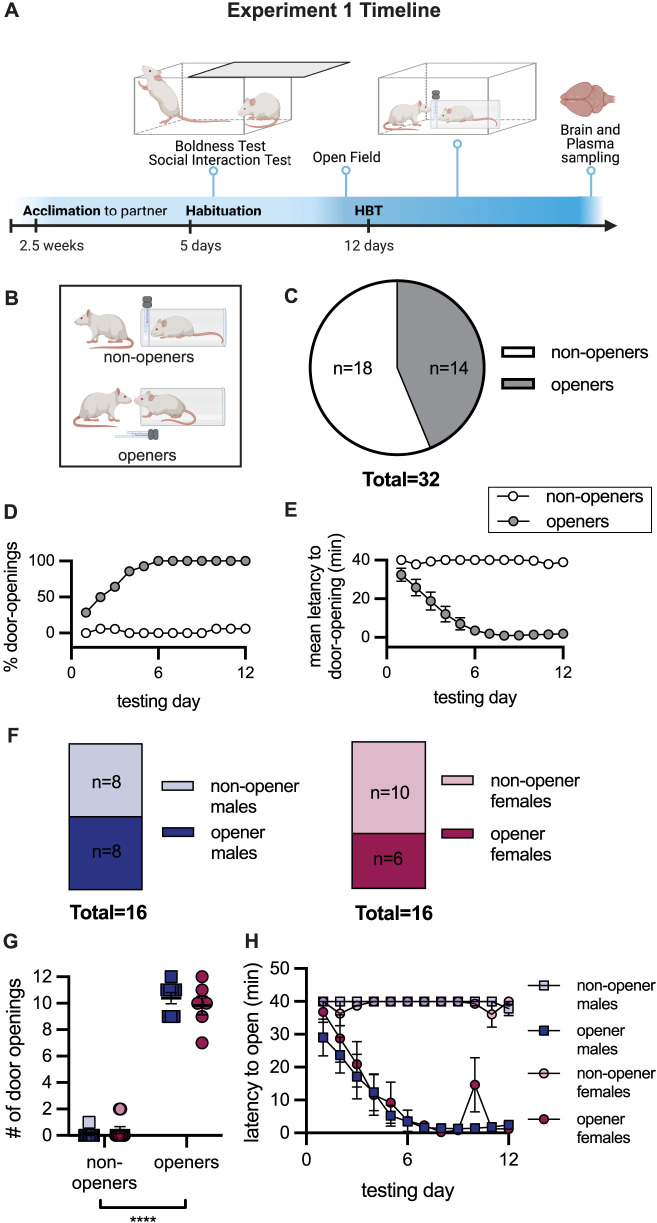
Adult helping behavior is similar in male and female rats. ***A***, Experimental timeline. During habituation, animals underwent a boldness test, a social interaction test, and an open field assay. The helping behavior test (HBT) consisted of 12 d of 1 h sessions. In the final session, brains and plasma were extracted for processing. ***B***, Animals were categorized into “openers” or “nonopeners” according to their behavior in the HBT. ***C***, Percent of openers across all animals: 43.75% (14/32) of rats became openers. ***D***, ***E***, For openers, helping behavior consisted of an increased % of door openings and decreased latency to open across testing sessions. ***F***, A similar percent of male rats (50%, 8/16) and female rats (37.5%, 6/16) became openers. ***G***, ***H***, The number of door openings across the 12 testing sessions did not differ by sex nor did the latency to open. Data are mean ± SEM.

After acclimation, rats underwent 5 d of habituation, during which they were accustomed to the experimenters, the experimental room, and the testing arena (Fig. 1*A*). This procedure was carried out to ensure that the only novel and stressful element during the HBT was the presence of a rat trapped inside the restrainer. On the first day, the rats were transported with their home cages to the testing room and remained there for 15 min without interruption before undergoing the boldness test. On Days 2–5, after the boldness test, the experimenters handled the rats for 5  min. Afterward, each pair was placed in the testing arena together (without the restrainer) for 30 min. Social interaction test (SIT) measures were obtained on Day 2 of habituation. On the 5th day, after the handling, the open field test (OFT) was conducted (Fig. 1*A*). Details for each test are found below.

##### Boldness test

The boldness test was used to reduce differences in door opening caused by the rats’ hierarchy within the pair or by individual traits such as curiosity and anxiety-like behavior (Ben-Ami Bartal et al., 2011). For 5 consecutive days, the metal grid top of the home cage was opened halfway, and the time it took for each rat to go to the open half, place its two front paws on the edge of the cage, and peek out was recorded. The test ended after 5 min if both rats did not peek out. The rat who peeked first at least 3 of the 5 d was assigned the “free rat” role and the other cagemate the “trapped rat” role. This protocol was conducted according to Ben-Ami Bartal et al. (2011). The mean latency to peek across the 5 d was calculated for each rat, as well as the difference, or delta, between the (future) free rat and the (future) trapped cagemate.

##### Social interaction scoring

Social interactions (SI) were measured prior to starting the HBT to characterize the social affiliation of each pair (Panksepp and Beatty, 1980; Kondrakiewicz et al., 2019). Each pair was placed together in the testing arena for 30 min. In the first 5 min, several social interaction measures were recorded for each pair, including the frequency and duration of sniffing, lying together, following, and climbing. Total social interaction time and number of social interactions were also measured on the first session of the HBT, during the 5 min after the door opened (whether by the free rat or by the trapped rat after the halfway door opening). For rats in Experiment 2 that underwent DREADD manipulations, a final 10 min social interaction test was conducted following the HBT. One group of animals received an intraperitoneal (i.p.) injection of 0.1 mg/kg deschloroclozapine (DCZ; Tocris Bioscience, catalog #1977-07-7) dissolved in 1% DMSO (i.e., DCZ group), while another received an injection of 1% DMSO diluted in 0.9% saline (i.e., saline group), 30 min prior to the social interaction test. In the mCherry control group, rats were tested three times in this 10 min SI test: following administration of DCZ, saline, or no injection (in counterbalanced order), to exclude nonspecific effects of DCZ or the injection itself. Experiments scoring behavior were blind to the animal's condition (opener or nonopener); in addition, separate individuals scored social behavior for Experiments 1 and Experiments 2 and 3.

##### Open field test (OFT)

The OFT was used to analyze the rats’ relative approach–avoidance behavior prior to starting the HBT (Walsh and Cummins, 1976; Saenz et al., 2006; Doron et al., 2012). Each rat entered the testing arena alone and underwent the OFT for 30 min. The following measures were taken: activity (measured by the number of centimeters the rat walked in the arena) and time in the center of the arena (the center was a quarter of the total arena size: 25 cm × 25 cm).

##### Helping behavior test (HBT)

For the HBT, a restrainer was added to the center of the testing arena (Harvard Apparatus). The restrainer, made of transparent Plexiglas (width, depth, height: 9 cm × 19.7 cm × 8.26 cm), had several slots so that communication between the rats was possible via sight, smell, hearing, and touch. The restrainer had a homemade door that could only be opened from the outside and, therefore, only by the free rat. There were two weights on the door, totaling 50 g. Because of the weights, a deliberate effort was required to open the door; a rat who wanted to open it and knew how to open it would succeed; however, the door would not open accidentally.

Following habituation, rats underwent 12 consecutive days of HBT testing (except Saturday; Fig. 1*A*). On each day, the free rat was placed into the arena once the restrainer with the trapped rat was set. The free rat had 40 min to open the door and release the trapped cagemate. Helping behavior is not shaped in any way by the experimenter; there is no prior training or exposure to the door prior to testing, in contrast to most operant lever pressing tasks. Even though the door was designed to be opened exclusively from the outside, some trapped rats managed to open it from the inside (*n* = 12, 37.5%). In this case, the trapped rat was returned immediately to the restrainer, and a blocker was added, preventing the trapped rat's access to the door. The blocker was then also used on all forthcoming days of testing. If the free rat opened the door, the experimenter removed the blocker immediately. After 40 min, if the free rat did not open the door, the experimenter opened the door halfway (to a 45° angle) for another 20 min. If there was a blocker, it was removed at this point. The partial opening encouraged the free rat to open the door and allowed the trapped rat to open it from the inside, avoiding learning helplessness. Only door opening that occurred by the free rat in the first 40 min was considered as door opening for analysis. At the end of the experiment, the number of total door openings was calculated for each pair. Based on previous studies (Ben-Ami Bartal et al., 2011, 2021), pairs in which the door was opened at least twice on the last 3 d were classified as “openers.” After classification, the percentage of rats that opened the door on each testing day was calculated per group. In addition, the average time to door opening was calculated [when the door was not opened, a 40 min (maximum test time) score was given]. In the first and last sessions, the following measures were recorded in the first 40 min: velocity, time in the corners, time around the restrainer, number of entries to the corners, and number of entries into the restrainer area. These measures were recorded only for rats that did not open the door in order to allow for statistical comparison.

For rats in Experiment 2 that underwent DREADD manipulations, HBT habituation and testing were similar to the HBT procedure described above. In addition to the regular handling, rats were handled for intraperitoneal injection restraining (with a needleless syringe). Rats that received the hM4D(Gi) virus were randomly assigned into two groups: the first was intraperitoneally injected daily with 0.1 mg/kg DCZ dissolved in 1% DMSO (i.e., DCZ group); the second group was injected daily with 1% DMSO diluted in 0.9% saline (i.e., saline group). Rats in the mCherry control virus group all received DCZ during the HBT testing. All injections were administered 30 min before the beginning of the HBT session. The trapped rats were alternated each day to avoid familiarity with the free rat.

##### Empty restrainer test

At the end of the HBT, all rats in Experiment 2 were tested in a control session, in which an empty restrainer was placed in the center of the arena, similar to the HBT sessions, and rats were allowed 10 min to explore. Injections were given 30 min prior to the test. Rats that received the hM4D virus were injected with either DCZ or saline based on their group assignment while rats in the mCherry control group were tested three times: following administration of DCZ, saline, or no injection (in counterbalanced order). Activity and location in the arena relative to the restrainer were measured.

#### Biological measures

##### Plasma and brain section collection

Three days after the paradigm ended, all rats were killed by rapid decapitation following a brief CO_2_ exposure. Both brains and plasma were collected from the free rats, while only plasma was collected from the trapped rats. Brains were snap-frozen on dry ice and stored at −80°C.

For plasma collection, trunk blood was first collected in EDTA tubes and centrifuged at 3,000 rpm, under 4°C for 15 min. Plasma was aliquoted and frozen at −80°C until CORT analyses. According to the manufacturer's protocol, plasma CORT concentrations were evaluated using an ELISA kit (EC3001, Assaypro).

Brains from 20 animals (10 openers, 10 nonopeners—5 of each sex) were sliced in coronal orientation on a cryostat. Tissue was obtained from each hemisphere using a 1-mm-diameter Miltex biopsy puncher (Bar Naor). One hemisphere's brain tissue punch was utilized for RNA sequencing, while the other hemisphere's punches were preserved for quantitative real-time PCR (qRT-PCR) analyses. Punches were taken directly while the brain was on the cryostat, and subsequent slices were used to confirm the thickness of the punch. Brain sections of the NAc and AI were collected, according to the following coordinates (Paxinos and Watson, 2006: NAc, AP 2.2–1.2 mm; AI, AP 3.2–1.2 mm). Tissue punches were stored in clean tubes and were immediately frozen on dry ice and stored at −80°C.

### Gene expression profiling

#### RNA sequencing (RNA-seq)

Isolated NAc and AI punches were subjected to genome-wide transcriptional profiling at the Technion Genomics Center and the University of California Los Angeles Social Genomics Core Laboratory, respectively.

For AI samples, RNA was extracted from 1-mm-diameter tissue punches of approximately 20 μg of frozen brain tissue (Qiagen RNeasy), assessed for suitable mass (RiboGreen), reverse transcribed to cDNA using a high-efficiency mRNA-targeted enzyme system (Lexogen QuantSeq 3′ FWD), and subsequently sequenced using Illumina NovaSeq instrument (Lexogen). Sequencing targeted at least 10 million sequencing reads per sample (achieved mean = 17.7 million), each of which was mapped to the mRatBN7.2 genome sequence (average 97.9% mapping rate) and normalized to transcripts per million using the STAR aligner.

For NAc samples, RNA was extracted from 1-mm-diameter tissue punches of approximately 20 μg of frozen brain tissue using the QIAcube Connect with an RNeasy micro kit (Qiagen) and assessed for suitable mass using an Agilent TapeStation System. The RNA integrity number values of all samples were in the range of 7.9–9.1, indicating a high quality. RNA-seq libraries were constructed using a NEBNext Ultra II Directional RNA Library Prep Kit (New England Biolabs). mRNA pull-down was performed using the Magnetic Isolation Module (New England Biolabs). After construction, the concentration of each library was measured using a Qubit (Invitrogen), and the size was determined using the TapeStation 4200 with a High Sensitivity D1000 kit (Agilent). All libraries were mixed into a single tube with equal molarity. RNA-seq data were generated using an Illumina NextSeq instrument (Lexogen) using P2 100 cycles (Read 1, 100; Index 1, 8; Index 2, 8). Quality control was assessed using FastQC (v0.11.5), and reads were trimmed for adapters, low quality 3`, and a minimum length of 20 using CUTADAPT (v1.12). Moreover, 100 bp single reads were aligned to a rat reference genome (Rattus_norvegicus.Rnor_6.0.faENSEMBL) and normalized to transcripts per million using the STAR aligner.

#### Quantitative real-time polymerase chain reaction (qRT-PCR)

Total RNA was first isolated with TRIzol (Thermo Fisher Scientific) and chloroform (Sigma-Aldrich). Subsequently, 25 ng of RNA per reaction was reverse transcribed into cDNA using the High-Capacity Reverse Transcription Kit with RNase Inhibitor (Thermo Fischer Scientific). The quantitative real-time polymerase chain reaction (PCR) was conducted using a Fast SYBR Green PCR Master Mix (Applied Biosystems) along with specific primers for *Oxtr* and GAPDH genes (HyLabs). To standardize gene expression levels, *Oxtr* expression was normalized to GAPDH, as the housekeeping reference gene. Product purity was validated through a melt curve analysis using applied biosystems hardware and software (QuantStudio Real-Time PCR Systems, Thermo Fisher Scientific), and gene expression analyses were determined using the comparative ΔΔCt (fold change) method.

### Immunohistochemistry

To examine neuronal activity associated with helping behavior, c-Fos expression was analyzed in a separate group of rats (Experiment 3). In brief, all animals performed the HBT experiment in similar conditions to the Wistar rats in Experiment 1, described above. However, several weeks before the habituation section, rats received a stereotactic injection of the retrograde tracer, Fluoro-Gold into the NAc (for complete methods, see Ben-Ami Bartal et al., 2021), and were allowed to recover before starting the behavioral tests. Moreover, on the last HBT session, the restrainer door was locked, and rats were perfused immediately after the session ended. An average of ∼24 slices per animal (mean = 24.38 ± 1.07 SEM; range, 15–28 slices) from atlas coordinates AP +5.2 to −8.3 (Paxinos and Watson, 2006) were analyzed with the automated software Brainways developed in-house (Kantor et al., 2025). The Brainways software allows automatic analysis of histological brain slices. Using this pipeline, slices were first matched to the atlas coordinates. Minor manual fine-tuning was then done by an experimenter blind to experimental conditions to optimize accuracy. At the next stage, the software automatically ran a cell detection algorithm, detecting c-Fos expression over the different slices. Then, cell density (number of c-Fos^+^ cells per 250 μm^2^) was calculated for each brain area. The normalization of cells per 250 μm^2^ ensured comparable quantification of cells across rats even if the area sampled was of a different size, and regions with less than three values per condition were excluded. Missing values were interpolated by the average value of all samples, for each condition.

### Viral injection validation

For the DREADD manipulation, immediately following the final testing session, rats were killed, and brains were obtained after perfusions with 100 ml of 1× PBS and 100 ml of 4% paraformaldehyde. Brains were cryosectioned at 40 µm, and slices were mounted and coverslipped with Vectashield HardSet Antifade Mounting Medium with DAPI (catalog #H-1500-10, Vector Laboratories), dried overnight, and stored at 4°C until imaging. The tissue was imaged at 10× using a widefield fluorescence microscope (Olympus IX83) for viral expression validation.

### Statistical analyses

#### RNA-seq analysis

The differential expression of each gene (DEG) was estimated with a standard linear statistical model relating log2-transformed transcript abundance values to measure individual status as opener versus nonopener while controlling for sex. A priori hypotheses were generated for key genes related to social behavior and reward, including for genes related to OXT, dopamine (DA), and corticotropin-releasing hormone (CRH) receptors, as well as genes associated with early immediate genes like *Fos*. Fold change analyses comparing openers and nonopeners were conducted on each of these genes.

We next applied a bioinformatic analysis of transcription factor binding motifs (TFBMs) in core promoter sequences of the differentially expressed genes (DEGs), using the Transcription Element Listening System (TELiS, http://www.telis.ucla.edu/) on all genes showing ≥1.5-fold differential expression in opener versus nonopener animals. A priori hypotheses were generated for key transcriptional regulators related to immediate-early genes and stress pathways, such as KROX, API, CREB, and SP1. For all bioinformatics analyses, standard errors were computed and estimated by bootstrap resampling of linear model residual vectors across genes (200 cycles); this controls for any statistical dependence among genes (Cole et al., 2005; Powell et al., 2013).

#### Task partial least square analysis

The multivariate task partial least square (PLS) analysis was based on brain regions’ c-Fos expression to identify optimal neural activity patterns that distinguished between the experimental groups (McIntosh et al., 1996; Mcintosh, 1999). Task PLS looks for latent variables (LVs) that explain a significant portion of the data variability. Through singular value decomposition, PLS produces a set of mutually orthogonal LV pairs. One element of the LV depicts the contrast, which reflects a commonality or difference between conditions. The other element of the LV, the relative contribution of each brain region (termed here “salience”), identifies brain regions that show the activation profile across tasks, indicating which brain areas are maximally expressed in a particular LV. Statistical assessment of PLS was performed by using permutation testing for LVs and bootstrap estimation of standard error for the brain region saliences. For the LV, significance was assessed by permutation testing: resampling without replacement by shuffling the test condition. Following each resampling, the PLS was recalculated. This was done 500 times in order to determine whether the effects represented in a given LV were significantly different than random noise. For brain region salience, reliability was assessed using bootstrap estimation of standard error. Bootstrap tests were performed by resampling 500 times with replacement, while keeping the subjects assigned to their conditions. This reflects the reliability of the contribution of that brain region to the LV. Brain regions with a bootstrap ratio >2.57 (roughly corresponding to a confidence interval of 99%) were considered as reliably contributing to the pattern. Missing values were interpolated by the average for the test condition. An advantage to using this approach over univariate methods is that no corrections for multiple comparisons are necessary because the brain region saliences are calculated on all of the brain regions in a single mathematical step. MATLAB code for running the task PLS analysis is available for download from the McIntosh lab website.

#### Network analysis

Network maps were created using a correlation matrix of c-Fos^+^ cells between all brain regions (using pairwise Pearson’s correlation coefficient calculations). Based on scale-free network characteristics described previously (Ben-Ami Bartal et al., 2021; Breton et al., 2022), only the top 10% correlations were used to produce the network graphs. Correlation values higher than the cutoff were set to 1 and the corresponding brain regions >1 were considered connected to the network. For more detailed methods, see Ben-Ami Bartal et al. (2021).

#### Additional statistical analyses and details

The variables “group” (openers, nonopeners), “drug condition” (Gi + DCZ, Gi + saline, mCherry + DCZ), and “sex” (male, female) were analyzed as between-subject variables, and the variables “role” (free, trapped) and “days of test” (1–12) were analyzed as within-subject variables. For several tests for the mCherry control group, “drug condition” (DCZ, saline, no injection) was considered a within-subject variable. A chi-square test of independence was used to analyze if there was a difference in the number of rats that became openers based on sex or experimental batch. Friedman's test was used to test for differences in the proportion of door opening across groups. All other measures were analyzed with analysis of variance (ANOVA) or *t* tests, as appropriate. Sidak's post hoc tests were used for multiple comparisons. Pearson’s correlations were used to identify relationships between social interaction measures and helping and c-Fos metrics. In all tests, the significance level was set at *p* < 0.05. Results are displayed and reported as means ± the standard error of the mean (SEM). Statistical analyses were conducted using SPSS (version 26), Prism (version 9; GraphPad Software), or MATLAB.

## Results

### Adult male and female rats behaved similarly toward trapped cagemates during the HBT, with approximately 50% of animals demonstrating helping

In Experiment 1, 32 pairs of adult male and female Wistar rats were tested in the HBT with trapped cagemates of the same strain and sex over a 2-week period (Fig. 1*A*). During the 1 h testing sessions, free rats could open the restrainer door thereby releasing their trapped cagemate. Based on their door-opening behavior, free rats tested in the HBT were classified as “openers” if they learned to release the trapped rat and demonstrated persistent door opening over consecutive testing sessions or “nonopeners” if they failed to do so (Fig. 1*B*; see Materials and Methods). Out of all pairs tested, 43.75% of rats were classified as openers (*n* = 14/32, mean door opening = 10.21 ± 0.36), and 56.25% of rats were classified as “nonopeners” (*n* = 18/32, mean door opening = 0.28 ± 0.16; Fig. 1*C*). For openers, the proportion of door openings significantly increased (Friedman's test: *p* < 0.0001, Fig. 1*D*), and latency to door opening decreased (*F*_(2.5,32)_ = 21.30, *p* < 0.0001, Fig. 1*E*) along the testing days, which was not the case for nonopeners (*p* > 0.05). A similar proportion of openers was observed for males and females, with 8/16 of males and 6/16 of females becoming openers (Fisher's exact test: *p* = 0.7224, Fig. 1*F*). A two-way ANOVA examining the effects of sex and opener status on the number of door openings identified a main effect of opener status (*F*_(1,28)_ = 656.5, *p* < 0.0001) but no main effect of sex and no interaction between sex and opener status (*p* > 0.05, Fig. 1*G*). Similarly, a mixed-effects model testing the effects of sex and opener status on door-opening latency across the testing days identified a main effect of time (*F*_(11,297)_ = 18.36, *p* < 0.001) and opener status (*F*_(1,28)_ = 474.3, *p* < 0.0001) and a significant interaction between time and opener status (*F*_(11,297)_ = 18.96, *p* < 0.0001), but no effect of sex and no interactions between sex and opener status (Fig. 1*H*). As there were no sex effects in these primary analyses, males and females were grouped together for all subsequent analyses. In sum, approximately half of the animals learned to open the restrainer by the end of testing, with no observed sex differences.

### Opener rats showed greater affiliation with their trapped cagemate

**Figure 2.**
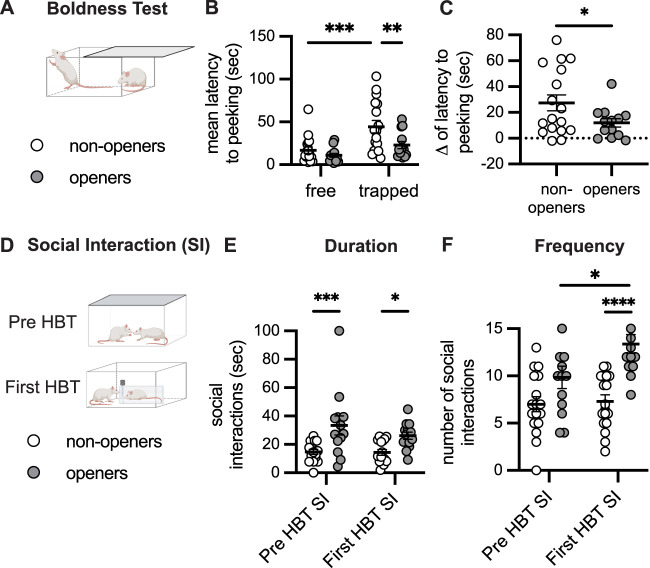
Social affiliation and boldness predict subsequent opening behavior. ***A***, Diagram of the boldness test conducted prior to the HBT. ***B***, Rats designated to become the “free” rat had a faster latency to peak during the boldness test than future “trapped” rats. ***C***, “Opener” pairs showed less of a difference in peeking latency within the cage than did “nonopener” pairs. ***D***, Social interaction was scored during habituation, prior to the HBT (pre-HBT), and on the first day of the HBT itself (first-HBT), following door opening. Openers showed increased duration (***E***) and frequency (***F***) of social interactions, both prior to the HBT and on the first day of HBT testing. For more details see Extended Data Figure 2-1. Data are mean ± SEM. *p < 0.05, **p < 0.01, ***p < 0.001, ****p < 0.0001.

We next assessed if behaviors prior to the start of the HBT could predict who would subsequently become openers. In a manner consistent with the criteria for the division into the free and trapped roles, in a two-way ANOVA, there was a main effect of rat role whereby in the boldness test, the (future) free rats peeked faster than the (future) trapped rats (*F*_(1,28)_ = 24.567, *p* < 0.0001; Fig. 2*A*,*B*). More importantly, there was also a main effect of opener status, whereby future openers (free and trapped rats) peeked faster than future nonopeners (*F*_(1,28)_ = 4.964, *p* = 0.034; Fig. 2*B*). There was also a trend for an interaction between rat role and subsequent opening status (*F*_(1,28)_ = 3.996, *p* = 0.055), though this did not reach statistical significance. In a post hoc test, among the (future) trapped rats, rats from the opener group peeked faster than those in the nonopener group (*p* = 0.026; Fig. 2*B*). However, among the (future) free rats, there was no significant difference between openers and nonopeners (*p* = 0.216; Fig. 2*B*). We next calculated the difference in peeking latency within each pair of cagemates (Fig. 2*C*). Here, there was a statistically significant difference between the future nonopener and opener groups (*t*_(23)_ = 1.287, *p* = 0.0389), with nonopeners showing a greater discrepancy in peeking time between the two cagemates (27.4 ± 6.3 s) relative to openers (11.9 ± 3.3 s; Fig. 2*C*). Although the opening phenotype was very robust on the individual level (Extended Data Fig. 2-1*A*), there were no group differences in either velocity (Extended Data Fig. 2-1*B*) or time spent in the center (Extended Data Fig. 2-1*C*) during open field testing, indicating that baseline movement and anxiety-like behavior are not likely explanations for these findings, nor do they predict subsequent opening.

10.1523/JNEUROSCI.0845-24.2025.f2-1 Figure 2-1 **Detailed door-opening and movement data. *A***) Mean latency to door-opening across testing sessions for each individual animal. ***B-C***) Velocity and time spent in the center of the 30-minute open field test did not differ across any condition. ***D***) Movement patterns for the free rat on day 1 of helping (including velocity, time and number of entries to the restrainer area and time and number of entries to the corner) did not differ between nonopeners and openers. ***E***) For non-openers, movement patterns were altered by day 12, with reduced velocity and entries into the restrainer zone, and more entries and time spent in the corners. Download Figure 2-1, TIF file.

Next, we looked at social interactions (SI), both prior to the HBT and on the first day of the HBT after the restrainer door had been opened either by the free rat (*n* = 4/32) or at the halfway point by the trapped rat (*n* = 28/32 rats; Fig. 2*D*). A two-way ANOVA comparing opener and nonopener groups at these two timepoints revealed a main effect of opener status on SI duration (*F*_(1,54)_ = 18.4, *p* < 0.001) with no effect of session (pre-HBT or Day 1 of HBT) and no interaction between them (Fig. 2*E*). Planned post hoc tests indicated that overall, compared with the nonopener pairs, the pairs from the opener group spent a greater amount of time interacting with one another, both prior to the HBT and on Day 1 of the HBT (*p* = 0.001 and *p* = 0.044, respectively). This indicates that rats that would subsequently become openers displayed more affiliative social interactions at baseline. A robust main effect of opener status was also found for SI frequency (*F*_(1,56)_ = 24.29, *p* < 0.001), as well as a main effect of session (*F*_(1,56)_ = 4.467, *p* = 0.039) and a trend toward an interaction between them (*F*_(1,56)_ = 3.201, *p* = 0.079; Fig. 2*F*). Planned post hoc tests indicated that openers had significantly more frequent social interactions than nonopeners on Day 1 of the HBT (*p* < 0.001); this effect was trending prior to the HBT (*p* = 0.06) but did not reach statistical significance. Furthermore, there was a significant difference in the frequency of interactions across sessions, with openers, but not nonopeners, showing an increased number of interactions on the first day of the HBT compared with the pretest session (*p* = 0.0241; Fig. 2*F*).

To further identify differences in motivational state between openers and nonopeners, movement patterns prior to door opening were also analyzed during the first HBT session. This analysis revealed that openers and nonopeners showed similar activity patterns around the trapped rat, including velocity, time spent in the arena corners, time around the restrainer, and number of entries to these regions (*p* > 0.05 for all measures; Extended Data Fig. 2-1*D*). This indicates a similar motivational state for rats tested with a trapped cagemate regardless of their subsequent pattern of helping behavior. Furthermore, while it is impossible to determine with certainty whether nonopeners failed to open the restrainer due to a lack of motivation or a lack of ability to learn the task, a significant reduction in their efforts and other-focused behavior was observed by the end of testing (Day 12–Day 1; Extended Data Fig. 2-1*E*) suggesting that nonopeners were less perseverant.

Thus, affiliative behavior, both prior to the HBT and during the first session that was expressed toward the released rat, was a better predictor of subsequent door opening than movement patterns around the trapped rat in the same session. Overall, this indicates that social interaction is a strong predictor of future opening behavior.

### Oxytocin receptor mRNA levels were elevated in the nucleus accumbens (NAc) of openers compared with nonopeners

**Figure 3.**
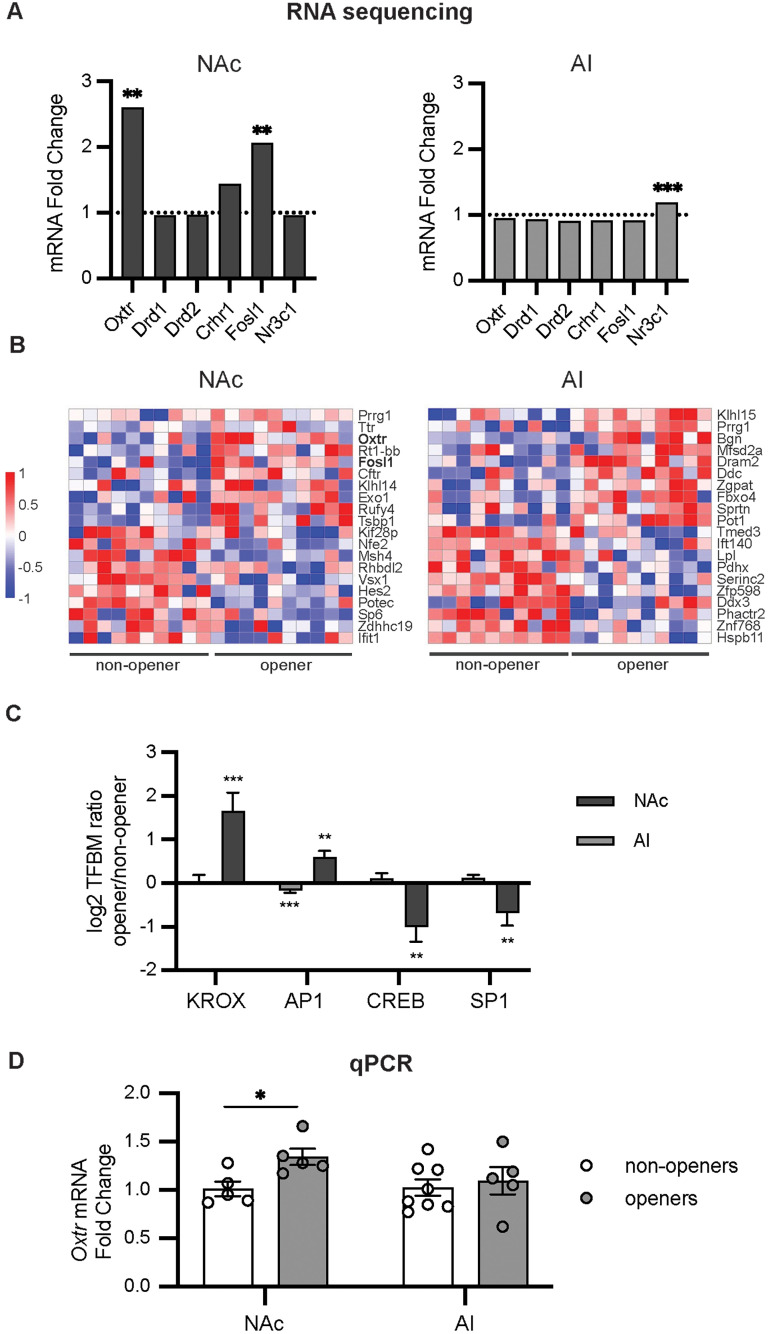
RNA analyses comparing openers and nonopeners. ***A***, RNA sequencing of a priori defined genes of interest in the NAc and AI. See Extended Data Table 3-1 for the list of genes. Increased oxytocin receptor and Fos gene expression levels were observed in the NAc of openers relative to nonopeners. Data are shown as a fold change, with 1 indicating no difference between openers and nonopeners and changes >1 indicating elevated levels in openers relative to nonopeners. ***B***, Top 10 upregulated and top 10 downregulated differentially expressed genes (DEGs) in the NAc and AI. Red indicates upregulation in openers relative to nonopeners, while blue indicates downregulation relative to nonopeners. See Extended Data Table 3-2 for the values of these genes. ***C***, Transcription factor binding motif (TFBM) prevalence in openers versus nonopeners. Positive numbers indicate increased prevalence in openers, while negative numbers indicate increased prevalence in nonopeners. ***D***, Quantitative real-time PCR (qRT-PCR) results of oxytocin receptor mRNA levels in the NAc and AI. Data are presented as a fold change with nonopeners having a mean of 1. *p < 0.05, **p < 0.01, ***p < 0.001.

To identify genome-wide transcriptomic differences that correspond with opening behavior in the HBT, we utilized RNA sequencing to measure the effect of opening behavior on gene expression in the NAc and AI; we focused on these two regions given their role in empathy (Wu and Hong, 2022), helping behavior (Ben-Ami Bartal et al., 2021), and social reward (Dölen et al., 2013; Dölen and Malenka, 2014; Fig. 3*A*). Based on a priori hypotheses, we first examined changes in genes related to oxytocinergic (*Oxtr*) and dopaminergic (*Drd1*, *Drd2*) signaling, as well as genes related to the stress axis (*Crhr1*, *Nr3c1*) and immediate-early gene (IEG) activity (*Fos1l*; Fig. 3*A*, Extended Data Table 3-1). In particular, given the known role of oxytocin in social reward (Dölen et al., 2013; Dölen and Malenka, 2014), we hypothesized that oxytocin receptor gene expression would be differentially expressed in openers and nonopeners. *Oxtr* expression was significantly upregulated in openers compared with nonopeners in the NAc (2.6-fold change, *p* = 0.006) but not in the AI (0.948-fold change, *p* = 0.463). No significant differences in DA receptor or CRH receptor gene expression were observed in either the NAc or AI of openers compared with nonopeners. However, a significant upregulation in *Nr3c1*, the gene encoding the glucocorticoid receptor, was found in the AI, but not in the NAc, of openers (1.19-fold change, *p* = 0.0289). Additionally, a marker for neuronal activation, the *Fosl1* gene, was significantly upregulated only in the NAc of openers (2.067-fold change, *p* = 0.0233; Fig. 3*A*). Together, this suggests that differences in OXT, but not DA or CRH, signaling in the NAc play a role in helping behavior.

10.1523/JNEUROSCI.0845-24.2025.t3-1 Table 3-1 **Genes of interest.** List of *a priori* genes analyzed in Figure 3. Download Table 3-1, DOCX file.

10.1523/JNEUROSCI.0845-24.2025.t3-2 Table 3-2 **Altered gene expression.** Top 25 statistically significantly up-regulated and down-regulated transcription factor binding motifs (TFBMs) in promoters of differentially expressed genes within the nucleus accumbens (NAc) and anterior insula (AI). Positive values indicate increased expression in openers relative to non-openers. Download Table 3-2, DOCX file.

RNA sequencing also allowed us to explore the relationship between opening behavior and transcriptome-wide gene regulation in these two regions. We conducted an analysis of differential gene expression (DEG) in NAc and AI samples from opener versus nonopener animals while controlling for sex. This analysis identified 463 of ≥1.5-fold DEGs in the NAc (226 upregulated and 237 downregulated) and 956 in the AI (593 upregulated and 363 downregulated). The top 10 upregulated and downregulated DEGs are shown in Figure 3*B*. Importantly, in the NAc, both *Oxtr* and *Fosl1* were in the top 10 upregulated genes found in openers. Of note, *Prrg1*, a gene encoding a vitamin K-dependent transmembrane protein, was the only gene significantly upregulated in both the AI and NAc of openers. Though the function of many of these genes is still being explored, together, these genes provide candidate targets that may contribute toward opening behavior.

Next, we applied a bioinformatic analysis of transcription factor binding motifs (TFBMs) in core promoter sequences of genes differentially expressed in the NAc or AI of opener versus nonopener rats (Fig. 3*C*) using the Transcription Element Listening System (TELiS; Cole et al., 2005). Two transcription factors, KROX and AP1, were looked at a priori given their association with immediate-early gene (IEG) activity (Dragunow, 1996; Kovács, 2008). This analysis showed a significant increase in KROX and AP1 activity in openers in the NAc (1.65 ± 0.418, *p* = 0.001, and 0.599 ± 0.139, *p* = 0.0001, respectively; Fig. 3*C*). However, in the AI, there was no significant change in KROX activity (0.028 ± 0.153, *p* = 0.85), while AP1 activity showed a small but significant decrease (−0.169 ± 0.049, *p* = 0.001; Fig. 3*C*). As both KROX and AP1 transcription factors are associated with IEG activity, the simultaneous elevation of these factors in the NAc suggests a higher level of neuronal activation in the NAc in rats with a history of opening. Furthermore, these findings align with the observed upregulation of the *Fosl1* gene in the NAc (Fig. 3*A*). In addition, both KROX and AP1 appeared in the top 25 upregulated TFBMs in the NAc (for a full table, see Extended Data Table 3-2). CREB was another key transcription factor of interest, given the CREB/ATF family's broader role in emotional regulation (Green et al., 2008) and prior work observing stress-associated upregulation of CREB in the NAc (Barrot et al., 2002; Muschamp and Carlezon, 2013). In the NAc, there was significantly decreased CREB activity for openers compared with nonopeners (−0.996 ± 0.352, *p* = 0.01), with no significant difference in the AI (0.108 ± 0.124, *p* = 0.38; Fig. 3*C*). Furthermore, ATF family transcription factors were among the top downregulated TFBMs in the NAc (Extended Data Table 3-2). Notably, SP1, a transcription factor associated with oxidative stress (Ryu et al., 2003), was significantly decreased in the NAc of openers (−0.687 ± 0.290, *p* = 0.02), with no significant difference in the AI (0.121 ± 0.0710, *p* = 0.09; Fig. 3*C*).

To validate our RNA-seq findings, we next performed qPCR to analyze *Oxtr* mRNA levels in the AI and NAc, using tissue from the same animals as the RNA-seq study (Fig. 3*D*). In line with the RNA-seq results, qPCR revealed a significant increase in *Oxtr* expression in the NAc (*t*_(8)_ = 2.91, *p* = 0.02) but not the AI (*p* > 0.05) of openers compared with nonopeners (Fig. 3*A*). In sum, across two different methods, we found substantial gene expression changes in the NAc, with increased *Oxtr* expression in animals with a history of opening behavior; this aligns with our previous knowledge of the NAc and its known role in helping behavior (Ben-Ami Bartal et al., 2021).

### Effect of chemogenetic inhibition of PVN oxytocin on helping behavior

**Figure 4.**
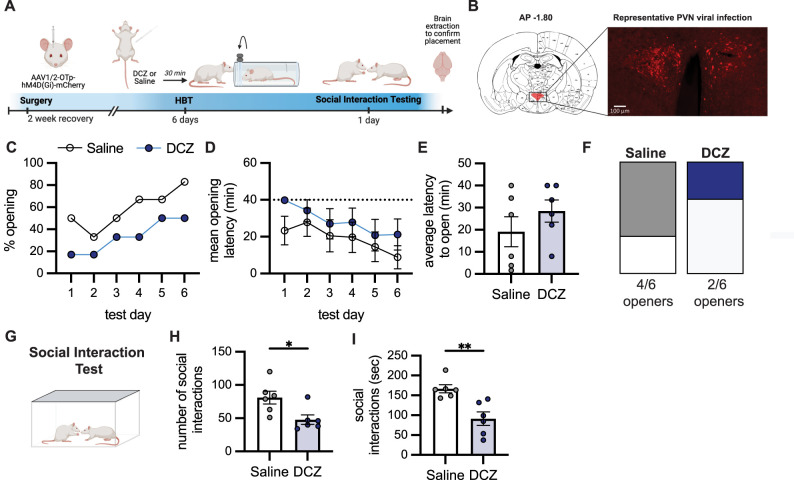
PVN OXT inhibition associated with reduced social interaction. ***A***, Experimental timeline. ***B***, Representative PVN viral infection from one rat at bregma −1.80. Scale bar, 100 μm. See Extended Data Figure 4-1 for the viral injection summary. Percent door openings increased (***C***) and latency to open decreased (***D***) across testing days for both saline (clear) and DCZ (blue) groups. The dashed line at 40 min indicates when experimenters opened the door halfway. ***E***, Average latency to open across the testing days. ***F***, Proportion of saline- (66.6%, 4/6) and DCZ-treated rats (33.3%, 2/6) that became openers. ***G***, A 10 min social interaction test was conducted after the HBT to test for effects of OXY inhibition on sociality. DCZ-treated rats showed fewer number (***H***) and duration (***I***) of social interactions relative to saline-treated rats.

In order to test the functional contribution of oxytocin to helping behavior, OXT^+^ neurons in the periventricular nucleus of the hypothalamus (PVN) were chemogenetically inhibited using DREADDs with an AAV under an oxytocin promoter (Experiment 2, see Materials and Methods, summary of injections at Extended Data Fig. 4-1). Thirty minutes prior to the HBT, animals received either an intraperitoneal injection of DCZ or saline (Fig. 4*A*,*B*). In this experiment, rats were tested in an abbreviated form of the HBT (6 d only). Here, Sprague Dawley (SD) rats were tested with unfamiliar rats of the same strain. Across the testing days, there was an increase in the % opening across both groups (DCZ or saline; Friedman's test *p* < 0.05) as well as a decrease in latency to open (DCZ, *F*_(5,25)_ = 4.826, *p* = 0.0032; saline, *F*_(5,25)_ = 15.41, *p* < 0.0001; Fig. 4*C*,*D*). There were no statistical differences between DCZ or saline animals across testing days, and although rats in the DCZ condition had a slower latency to open on average (DCZ, 28.5 ± 5.0; saline, 19.1 ± 6.8), this effect was not statistically significant (Fig. 4*E*). Overall, most rats in the saline condition (*n* = 4/6, 66.6%) learned to open the restrainer by the end of the experiment, consecutively opening on the last 2 d of testing. In the DCZ-treated rats, a minority of the oxytocin-inhibited animals (*n* = 2/6, 33.3%) became openers (Fig. 4*F*). While this difference is not statistically significant, the effect of PVN OXT inhibition on prosocial behaviors warrants further investigation.

10.1523/JNEUROSCI.0845-24.2025.f4-1 Figure 4-1 **Injection summary.** Viral injection summary in the PVN across all 12 rats for Experiment 2, including the hMRD(Gi) rats ***A***, and the mCherry control rats ***B***. Download Figure 4-1, TIF file.

10.1523/JNEUROSCI.0845-24.2025.f4-2 Figure 4-2 **Control conditions for viral manipulation. *A)*** Illustration of HBT set up. Percent-door openings increased)***B***) and latency to open decreased)***C***) across testing days for all three experimental groups. The dashed line at 40 minutes indicates when experimenters opened the door halfway. ***D***) Average latency to open across the testing days. ***E***) Proportion of mCherry-DCZ rats (60%, 3/5) that became openers. ***F***) A 10-minute social interaction test was conducted after the HBT in the mCherry-expressing rats to test whether DCZ administration or injection stress impacted sociability. No differences were observed in the duration (***G***) or number of social interactions (***H)***. ***I***) An Open Field Test was conducted prior to the HBT and without any pharmacological treatment to assess for effects of viral expression on activity measures. No differences were observed across the three conditions in the total duration in center (***J***) total distance moved ***(K***) and the frequency of entries into the center (***L***) ***M***) A 10-minute empty restrainer test was conducted after the HBT to test for an effect of OXT inhibition on non-social activity levels. No effect was observed in frequency around the restrainer in the hM4D-Gi groups (***N)*** or mCherry conditions (***O)*** nor was there differences in the cumulative duration spent around the restrainer (***P,Q)*.** Download Figure 4-2, TIF file.

We next asked whether oxytocin inhibition influenced social interaction in the HBT paired animals. Drug injections (DCZ or saline) were given 30 min prior to a 10 min social interaction test, where animals were freely allowed to move and interact in the arena (Fig. 4*G*). As with Experiment 1, 5 min of social interaction was scored. This analysis revealed that DCZ-treated rats showed reduced number and duration of social interactions with the previously trapped conspecific compared with saline-treated controls (number, *t*_(10)_ = 2.76, *p* = 0.02; duration, *t*_(10)_ = 3.83, *p* = 0.003; Fig. 4*H*,*I*). Thus, PVN OXT inhibition significantly reduced affiliative behavior.

An additional cohort of rats was injected with a control virus lacking the chemogenetic receptor to assess potential baseline deficits due to viral expression. No differences were observed in the opening behavior (Extended Data Fig. 4-2*A*–*E*), social interactions (Extended Data Fig. 4-2*F*–*H*), or baseline motor activity, as assessed in an OFT (Extended Data Fig. 4-2*I*–*L*). In addition, an empty restrainer control session was conducted at the end of each experiment, to test whether OXT inhibition impacted nonsocial activity levels. DCZ administration did not affect activity patterns around the empty restrainer in either the hM4D(Gi) or mCherry conditions (Extended Data Fig. 4-2*M*–*Q*), suggesting the specific involvement of PVN OXT neurons in a social context.

### Increased c-Fos was observed in social neural networks of openers compared with nonopeners

**Figure 5.**
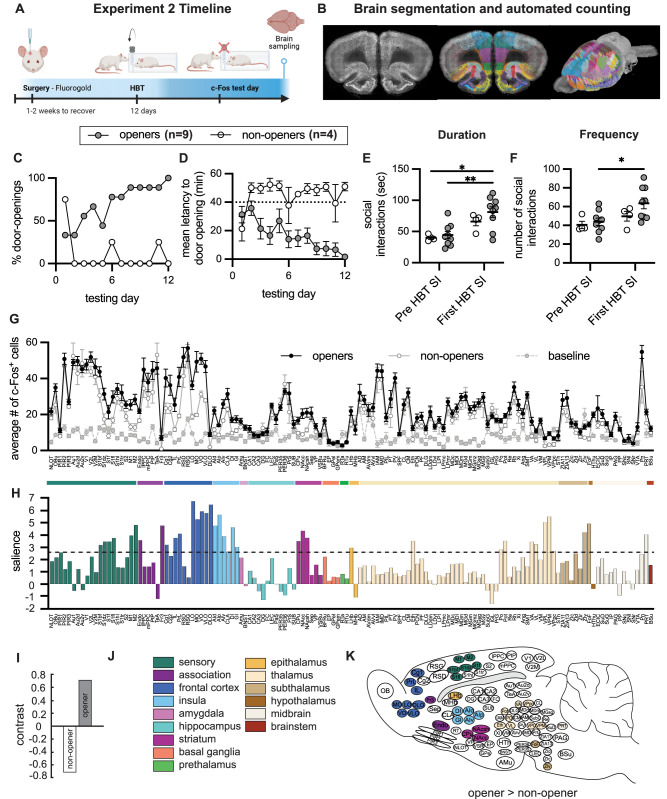
Brain-wide patterns of neural activity in openers. ***A***, Experimental timeline. ***B***, Representation of c-Fos analysis pipeline, following a previous study (Kantor et al., 2025). See the full list of regions analyzed in Extended Data Table 5-1. ***C***, ***D***, Percent door openings increased and latency to open decreased across testing days for openers. ***E***, ***F***, Duration and frequency of social interactions prior to and on the first day of the HBT. ***G***, Number of c-Fos^+^ cells per 250 μm region (mean ± SEM) for opener, nonopener, and baseline rats. See Extended Data Figure 5-1 for the boxplots of all regions. ***H***, Task partial least square (PLS) analysis. A history of opening in the HBT was associated with increased activity in multiple brain regions compared with all other conditions. Regions that cross the dashed significantly (*p* < 0.05) contributed to this pattern. ***I***, Openers and nonopeners showed distinct patterns of neural activity (PLS contrast). ***J***, Legend of brain region categories coded by color. ***K***, Diagram of rat brains showing regions significantly more activity in openers relative to nonopeners.

In order to map the neural circuits activated during the HBT in openers and nonopeners, the immediate-early gene c-Fos was analyzed across the brain in a separate group of adult SD male rats (*n* = 13) tested with a trapped SD cagemate for 2 weeks (Experiment 3, Fig. 5*A*). On the final testing session, the restrainer was latched shut, and c-Fos^+^ cells were measured as an index of the neural activity associated with an hour of being in the presence of a trapped cagemate (Fig. 5*B*). This strategy has previously been useful for us and others for describing neural networks involved in complex activity (Wheeler et al., 2013; Vetere et al., 2017; Ben-Ami Bartal et al., 2021; Breton et al., 2022). Here, across our 13 pairs, similar to Experiment 1, most rats (*n* = 8/13) exhibited robust door opening for trapped cagemates (average learning day, 5.13 ± 0.9) and were classified as “openers.” Additionally, one rat opened on 2 consecutive days, as well as on the final testing day, and was also categorized as an “opener”; thus, there were nine openers in total (*n* = 9/13, 69.3%). Several rats (*n* = 4/13, 30.7%) rarely opened the restrainer and did not show consecutive door-opening behavior; these were classified as “nonopeners” (Fig. 5*C*,*D*).

10.1523/JNEUROSCI.0845-24.2025.t5-1 Table 5-1 **Brain region list.** Detailed list of 137 brain regions analyzed and used in Figures 4-5. Brain region abbreviations and full names, organized by presentation and brain region category. Download Table 5-1, DOCX file.

10.1523/JNEUROSCI.0845-24.2025.f5-1 Figure 5-1 **Visualization of c-Fos data.** Box-plots showing c-Fos data for non-openers (red), compared to openers (green), and baseline animals (blue) by brain region. Download Figure 5-1, TIF file.

10.1523/JNEUROSCI.0845-24.2025.f5-2 Figure 5-2 **Brain pattern of opener and non-openers contasted with baseline. *A)*** Partial least square (PLS) task analysis, including the baseline condition. Animals that underwent the HBT showed increased activity in nearly all brain regions compared to an untested baseline. Regions that cross the dashed lines significantly (p < 0.05) contributed to this pattern. ***B)*** PLS contrast graph: openers and non-openers showed distinct patterns of neural activity, and contrasted significantly with the brain-wide activity pattern observed in the baseline condition ***C***) Legend of brain region categories coded by color, as seen in the main figure. Download Figure 5-2, TIF file.

As in Experiment 1, social interactions (SI) both prior to the HBT (pre-HBT) and on the first day of door opening (first-HBT) were scored. Though a two-way ANOVA comparing opener and nonopener groups at these two timepoints found no effect of opener status on SI duration and no interaction between opener status and session (*p* > 0.05), there was a main effect of session (*F*_(1,22)_ = 14.36, *p* = 0.001; Fig. 5*E*), with greater interaction duration on the first session of the HBT relative to the pretest session. Planned post hoc tests indicated that the pairs from the opener (but not the nonopener) group spent a greater amount of time interacting with their cagemate on Day 1 of the HBT compared with the baseline session (*p* = 0.004). Similar patterns were observed with SI frequency (Fig. 5*F*); there was a main effect of session (*F*_(1,22)_ = 6.45, *p* = 0.019), but neither an effect of opener status nor an interaction (*p* > 0.05). As with duration measures, post hoc tests indicated that openers (but not nonopeners) had significantly more frequent social interactions on Day 1 of the HBT relative to the pretest day (*p* = 0.03). This increase in affiliative interactions between openers (but not nonopeners) after releasing the trapped rat may be reflective of a reaction to the trapped rat's release rather than social contact alone. This is in line with an interpretation of the postrelease affiliative behavior as consolation or increased empathic sensitivity in openers. Overall, although no there were no statistically significant differences between SI in openers and nonopeners, the data demonstrate a similar pattern as observed in Experiment 1, with mean SI duration and frequency higher in the opener group relative to the nonopeners (especially on the first day of the HBT).

Using an in-house software (Kantor et al., 2025), c-Fos^+^ was quantified in 24 slices per rat on average, and the number of c-Fos^+^ cells was compared between openers and nonopeners as well as an undisturbed baseline group (*n* = 8; Fig. 5*B*,*G*; see Materials and Methods for details). In total, 137 regions were analyzed (Extended Data Table 5-1 displays all regions; see Extended Data Fig. 5-1 for the boxplots per region, including the baseline controls). In order to compare activity levels across regions, c-Fos^+^ cell numbers were normalized to a standard area of 250 µm^2^. Analysis of brain-wide c-Fos^+^ patterns using a multivariate task partial least square (PLS) approach (Fig. 5*H*–*K*; McIntosh et al., 1996; Mcintosh, 1999) found a significant latent variable (LV) for the contrast between c-Fos expression across the brain of openers and nonopeners (LV, *p* < 0.05; Fig. 5*I*). This reflects higher brain-wide c-Fos^+^ cell numbers in the openers compared with the nonopeners (*t* test, *t* = 7.26, *p* < 0.0001, Fig. 5*G*). Brain-wide activity for both openers and nonopeners was also significantly higher than the untested baseline condition (PLS: LV, *p* < 0.0001; Extended Data Fig. 5-2*A*–*C*), replicating prior work (Ben-Ami Bartal et al., 2021). Bootstrapping and permutation tests were then used to discover the pattern of neural activity associated with the contrast between openers and nonopeners (Fig. 5*H*, see Materials and Methods). Multiple brain regions significantly contributed to this contrast, including primary and secondary sensory regions such as somatosensory, motor, and olfactory cortices (Fig. 5*H*, green). Additionally, the orbitofrontal regions, anterior cingulate cortex, mediofrontal regions [infralimbic (IL), prelimbic (PrL)], insula, claustrum, lateral habenula (Lhab), ventral and posterior thalamic nuclei, subthalamic regions, and midbrain regions all contributed to this contrast (Fig. 5*H*). c-Fos in the orbitofrontal cortex (OFC) contributed most strongly to the contrast between openers and nonopeners, suggesting that this region in particular plays a key role in predicting helping behavior.

This analysis indicates that in the presence of a trapped cagemate, openers demonstrated significantly increased activity in a dispersed network of brain regions that have previously been associated with empathy and prosocial motivation (Wu and Hong, 2022), providing further support for the idea that this network supports a prosocial response toward conspecifics in distress. In addition, this analysis expands on previous work to include new brain regions and indicates a role for the IL, zona incerta, and specific thalamic areas such as the posterior nucleus, in prosocial motivation (Fig. 5*K*). Finally, as opposed to previous findings comparing ingroup and outgroup conditions (Ben-Ami Bartal et al., 2021), here we did observe significantly increased activation in the anterior insula (AI) and anterior cingulate cortex (ACC) of openers compared with nonopeners (Fig. 5*K*). In addition, a network analysis of the openers’ c-Fos identified the ACC as part of a centrally located cluster (Extended Data Fig. 5-3). Notably, the ACC was strongly connected to core regions of the prosocial behavior network, including the NAc shell and core, septum (Sep) prefrontal regions (IL, PrL), and the VTA and periaquaductal gray (Extended Data Fig. 5-3). Together, these data suggest the role of key prosocial brain regions in predicting individual variability in helping behavior.

10.1523/JNEUROSCI.0845-24.2025.f5-3 Figure 5-3 **Network analysis of openers.** In order to identify the functional connectivity involved specifically in adult helping behavior, a network analysis was conducted based on correlations between c-Fos + cells in all brain regions of helper (opener) rats. ***A)*** An inter-region Pearson’s pairwise correlation matrix. Red indicates a positive correlation, blue indicates a negative correlation. Four major clusters were identified (blue, yellow, green and pink). Coloring according to brain region group (see Figure 3I or Figure 5-2 for legend), and according to the network map shown below. ***B)*** Network graph for openers. Solid lines connecting regions denote the top 10% of positive correlations. Cluster 1 (blue) was composed mainly of sensory regions (auditory, visual, motor, & somatosensory cortices), as well as the insular cortex, some hippocampal regions (DG, CA3, perirhinal), the medial geniculate and the caudate and putamen (CPu). Cluster 2 (yellow) was composed of core regions of the prosocial response network described previously (Ben-Ami Bartal et al., 2021; Breton et al., 2022), including the NAc shell and core, OFC (VO, MO, VLO, LO, DLO), AI (Aid, AIv), PrL, claustrum, amygdala, lateral and medial habenula, ventral pallidum, olfactory regions, basal forebrain, and the frontal association area. Cluster 3 (green) included mainly thalamic regions, the periaqueductal grey (PAG) region, and regions of the zona incerta (ZI). Lastly, Cluster 4 (pink) included frontal cortex regions such as the ACC and IL, the retrosplenial cortex (RSG), some hippocampal regions (CA1, CA2, SUB), the septum, BNST, thalamic & subthalamic nuclei, hypothalamic regions, as well as regions of the midbrain (substantia nigra (SN) & ventral tegmental area (VTA)) and brainstem. This cluster was most centrally located on the network and served as a connection point between the three other clusters. Download Figure 5-3, TIF file.

### Social interactions correlate with latency to help and with c-Fos measurements

**Figure 6.**
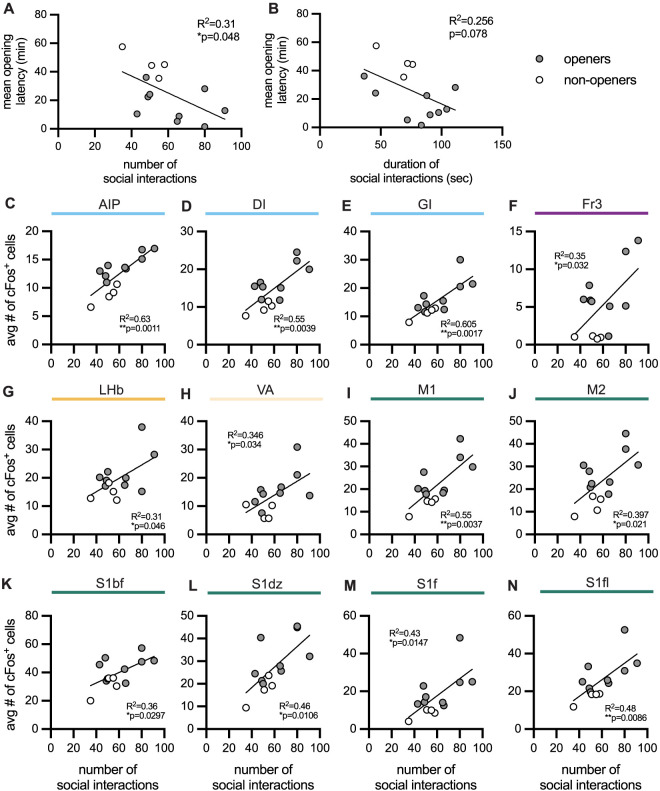
Correlations between social interaction and HBT behavior and brain activity. ***A***, ***B***, The number and duration of social interactions on the first day of the HBT correlated with the mean opening latency across all test days, with rats that showed more social interactions ultimately helping release the trapped rat faster. See Extended Data Table 6-1 for details. ***C***–***N***, Social interaction frequency was positively correlated with c-Fos levels in 12 of the 36 regions that had been found to contribute to the contrast between openers and nonopeners including several regions of the insula association cortex, lateral habenula, and midbrain, as well as motor and somatosensory regions. In all graphs, openers are shown in gray, and nonopeners in white.

In both Experiments 1 and 3, animals that ultimately became openers demonstrated higher levels, on average, of social interaction on the first day of the HBT. Here, Pearson's correlations were used to determine whether social interactions on the first HBT day predicted overall measures of helping. The frequency, but not duration, of social interactions on the first HBT day was significantly correlated with the mean latency to open across the testing period (frequency, *r* = −0.557, *p* = 0.048; duration, *r* = −0.506, *p* = 0.078; Fig. 6*A*,*B*) with faster latencies to open associated with a greater number of social interactions. As the frequency of social interactions was a predictor of helping behavior, we next assessed whether the number of social interactions on the first day of the HBT was associated with c-Fos levels on the final day of testing, focusing on brain regions that significantly contributed to the contrast between openers and nonopeners (Fig. 6*H*–*K*). Twelve of the 36 regions showed significant positive correlations (all at *p* < 0.05) between the social interaction number on the first HBT day and c-Fos on the final test day (Fig. 6). These included insula (Fig. 6*C*–*F*), association (Fig. 6*D*), thalamus and epithalamus regions like the lateral habenula (Fig. 6*G*,*H*), and sensory and motor regions (Fig. 6*I*–*N*). Importantly, c-Fos did not correlate with social interaction measures prior to the HBT (in any brain region), indicating that social interaction following the first release, but not baseline social interaction, was predictive of neural activity on the final test day. A table with all correlations and statistics can be found in Extended Data Table 6-1.

10.1523/JNEUROSCI.0845-24.2025.t6-1 Table 6-1 **Correlations of ROIs with social behavior.** The 36 brain regions that significantly contributed to the contrast between openers and non-openers were tested for correlations with social interaction on the first day of the HBT. Brain region category, region abbreviation and Pearson’s correlations are reported here, with statistically significant correlations in bold. Download Table 6-1, DOCX file.

## Discussion

Prosocial helping is observed across a wide range of species; however, even within the same social context, helping does not always manifest. These experiments took advantage of variability within a rodent model of helping behavior to explore behavioral and molecular changes that correspond with helping. Adult Wistar rats were tested with conspecifics of the same strain, a condition that typically elicits prosocial motivation (Ben-Ami Bartal et al., 2014). In this cohort, almost half of the animals demonstrated door opening, with no differences across sexes. Social interactions measured both prior to and on the first day of restrainer testing differentiated openers from nonopeners, with greater affiliative behaviors observed in animals that ultimately learned to open the restrainer. *Oxtr* expression, hypothesized to play a role in these differences, was quantified in the NAc and AI, two key regions of the prosocial brain network (Ben-Ami Bartal et al., 2021; Breton et al., 2022). Gene expression analyses identified elevated *Oxtr* expression in the NAc, but not AI, of openers and elevated glucocorticoid receptor gene expression levels in the AI, but not the NAc. Brain-wide measurements of c-Fos in a cohort of adult Sprague Dawley rats indicated heightened neural activity for openers relative to nonopeners in the prosocial brain network, including more activation in NAc, prefrontal, insular, and sensory regions. Importantly, neural activity markers in many of these brain regions were positively correlated with social interactions on the first day of restrainer testing. In sum, these findings indicate that affiliative behavior in dyads was associated with probability of helping and activation in the prosocial brain network.

Here, in Experiment 1, approximately half of the animals learned to open the restrainer by the end of testing. These numbers are somewhat lower than the previously observed proportions of openers (∼70% openers for cagemates; Ben-Ami Bartal et al., 2011). This difference may be driven by rat strain, as Wistar rats were used for Experiment 1 and prior work has been conducted using Sprague Dawleys. Importantly, the larger proportion of nonopeners in this experiment allowed us to more closely examine both the behavioral and neural differences between openers and nonopeners within the same social condition (i.e., tested with cagemates of the same strain). Notably, sex was also considered as a biological variable within our study design.

Across multiple experiments, less helping was observed in dyads that exhibited fewer social interactions. This finding is in line with human literature; prosocial motivation is influenced by affiliation (Wilbanks et al., 2005), and prosocial behavior is more likely to be extended to closely affiliated others (Batson, 2011). While affiliative behavior has been demonstrated to influence helping in primates (Cronin, 2012), to our knowledge, the current study is the first to examine this in rats.

The association of affiliation with helping may indicate that, in highly affiliated pairs, increased social reward experienced from postrelease contact motivates door opening. Yet previous studies have demonstrated that social contact is not required for helping to occur (Ben-Ami Bartal et al., 2011; Sato et al., 2015; Cox and Reichel, 2020). Thus, although social contact may play a role in motivating helping, it is unlikely to completely explain this behavior. Alternatively, rats in affiliated pairs may find the conspecifics’ distress more salient or place a higher value on alleviating their distress, an effect that could be mediated by OXT signaling. For instance, pup retrieval has been shown to depend on an OXT-driven increase in synchronization in the auditory cortex (Marlin et al., 2015; Carcea et al., 2021). Additionally, in a recent study, OXT receptor antagonism in the ACC delayed, but did not fully suppress, helping behavior in a variation of the HBT (Yamagishi et al., 2020).

Here, *Oxtr* expression was elevated in the NAc of opener rats relative to nonopeners, in line with evidence for the role of NAc *Oxtr* in social approach (Yu et al., 2016; Williams et al., 2020), affiliative behavior (Ross and Young, 2009; Burkett et al., 2016; King et al., 2016), and social reward (Dölen et al., 2013; Hung et al., 2017). No difference was observed in the AI, mirroring prior work showing that *Oxtr* expression in the NAc, but not AI, predicts social attachment (King et al., 2016). However, AI OXT blockade has been shown to reduce approach to a distressed conspecific (Rogers-Carter et al., 2018). *Drd1* and *Drd2* genes were also not different between openers and nonopeners in these regions, despite their reported role in social play (Manduca et al., 2016), social attachment (Gingrich et al., 2000), and other prosocial behaviors (Walsh et al., 2023). Here, RNA-seq identified additional genes enriched in the NAc and AI of openers, providing targets for future investigation. Analyzing TFBMs in promoters of DEGs revealed that CREB target genes were significantly downregulated in the NAc of the opener group. CREB is upregulated in the NAc following stress and is associated with depressive-like symptoms and dysregulation in motivated behavior (Barrot et al., 2002; Carlezon et al., 2005; Muschamp and Carlezon, 2013; Manning et al., 2017). Thus, CREB downregulation in openers might suggest that helping behavior is protective against a depressive-like phenotype. Additionally, KROX and AP1 activity were elevated in the NAc of openers. As these factors represent immediate-early gene activity, these findings are congruent with the increased levels of NAc c-Fos in openers. Overall, we found that gene expression and transcription factor changes in the NAc, rather than the AI, were related to helping behavior, and OXT signaling in the NAc is a potential target for future manipulations aimed at increasing helping behavior.

As a first step to target OXT signaling across the brain, in a cohort of rats, we chemogenetically inhibited OXT in the PVN during the HBT. Though OXT inhibition slowed door opening and fewer DCZ-treated rats ultimately became openers compared with saline-treated rats, this result was not statistically significant. Despite this result not being significantly different than saline-control, DCZ-treated rats showed lower levels of helping than previously observed, with only two rats becoming openers. However, in a follow-up test of social interaction, DCZ reduced both the frequency and duration of interactions with the previously trapped rat, in line with prior work supporting the critical role of OXT in sociality (Carter et al., 2008; Resendez et al., 2020). Combined with the findings on OXTr in the NAc from the other experiments, the NAc is a likely target for future manipulations.

In prior work, we tested a multitude of control groups, both behaviorally and for c-Fos levels; this included an untested baseline condition, a group exposed tested with a food reward (chocolate chips), and a group tested in a brief version of the paradigm across 3 d, among others (Ben-Ami Bartal et al., 2021). We previously reported that animals tested in the HBT show elevated c-Fos levels across the prosocial brain network relative to the baseline group (Ben-Ami Bartal et al., 2021). Here, we replicate those findings with new groups using a novel, in-house open-source freeware, Brainways (Kantor et al., 2025).

Importantly, here we identified increased activity in the previously outlined prosocial brain network (Ben-Ami Bartal et al., 2021) for openers compared with nonopeners, including in the NAc, AI, OFC, and sensory regions. This finding adds validity to our previous observations of increased NAc activity for trapped ingroup members compared with outgroup members and further suggests that NAc activity is predictive of helping.

The OFC, a region known for its role in goal-directed, value-based, and effort-related responding (Wallis, 2007; Münster and Hauber, 2018; Rudebeck and Rich, 2018; Woon et al., 2020), has consistently arisen as a key region active in the HBT. While prior work found the medial OFC to be uniquely active in rats tested with ingroup relative to outgroup members (Ben-Ami Bartal et al., 2021), here, elevated c-Fos was observed across all OFC subregions in openers tested with the same strain, providing support for the involvement of this region in helping behavior and pointing to valuation processes being involved in helping.

The sensory and insular cortex regions also showed heightened c-Fos activity in openers relative to nonopeners. This difference may indicate heightened responsivity to the trapped cagemate, which is associated with an increased likelihood of helping. While past work considered activation of these regions to be common across all conditions of the HBT (regardless of group identity; Ben-Ami Bartal et al., 2021), here we clearly observe different sensory and insula activity for the same social condition. This suggests that both sensory and insular processing differences are related to the motivational state of the free rat rather than the biological identity of the trapped rat. Interestingly, activity in the insula and sensory regions was positively correlated with social dynamics on the first day of the HBT, indicating that neural activity of these regions can be influenced by other parameters, including the relationship between the two animals.

The current study has several limitations. Door opening is a complex task, recruiting multiple systems in addition to prosocial motivation, such as motor skills, learning, and memory. When rats open the restrainer, it has a clear goal-directed outcome, while failure to open the door is ambiguous, as it may stem from a lack of motivation or failure to learn the task. Thus, it is possible that some nonopeners in this experiment do have prosocial motivation but are unable to release the trapped rat. Future experiments will be needed to rule out this confounding factor, for instance, by testing rats for general traits before HBT exposure. An additional caveat is that Experiment 1 was conducted with Wistars and Experiments 2 and 3 with Sprague Dawleys. The observed changes in gene expression should be tested with a Sprague Dawley strain in the future, to ensure that findings translate across multiple rat strains. Additionally, although female rats were included in Experiment 1, Experiments 2 and 3 used only males. It remains possible that, though behavioral manifestations of helping were similar across sexes, the biological mechanisms driving this behavior are distinct. Though gene expression changes were similar across sexes, future work will need to analyze neural activity in both; this is especially critical as others have reported neural but not behavioral differences in helping behavior in male and female rats (Cox et al., 2024). The present study was also underpowered to examine functional connectivity maps and the difference between openers and nonopeners. Of particular interest, future work could test whether ACC regions are functionally connected to the NAc only in openers (and not nonopeners), supporting prior work (Ben-Ami Bartal et al., 2021). In general, future work including a larger number of nonopeners will be needed to support the findings of the current study. Moreover, there was some variability in the number of slices quantified per rat. While cell count was normalized to an area of 250 µm^2^, it is possible that some variability could have been reduced by standardizing this parameter. Another limitation of this study is the impossibility of knowing whether differences in gene expression occurred prior to helping, or due to experience in the HBT. Future work can test for a causal role of *Oxtr* through genetic manipulation and should examine *Oxtr* expression across additional brain regions. Finally, it is impossible to dissociate trait sociability from dyadic interactions, and thus, future studies should aim to control for trait sociability prior to testing to assess the impact of dyadic dynamics on helping behavior.

In sum, these data suggest that social affiliation plays a critical role in variability in prosocial helping behavior. While it is well supported that mammals tend to help those that are familiar/ingroup members, these findings suggest that relationship strength is predictive of helping even within the ingroup. Furthermore, this work explores neural variability and brain-wide changes associated with prosocial behavior, implicating NAc *Oxtr* levels in contributing toward prosocial helping and highlighting a relationship between sociality, helping behavior, and neural activity. Together, this work holds important implications for understanding differences in prosocial motivations and provides targetable mechanisms for future study.
