# Supplementary figures and images for: Neural and Behavioral Correlates of Individual Variability in Rat Helping Behavior: A Role for Social Affiliation and Oxytocin Receptors
Source: J Neurosci. 2025 Apr 28;45(22):e0845242025. doi: 10.1523/JNEUROSCI.0845-24.2025 (PMC12121707; doi:10.1523/JNEUROSCI.0845-24.2025)

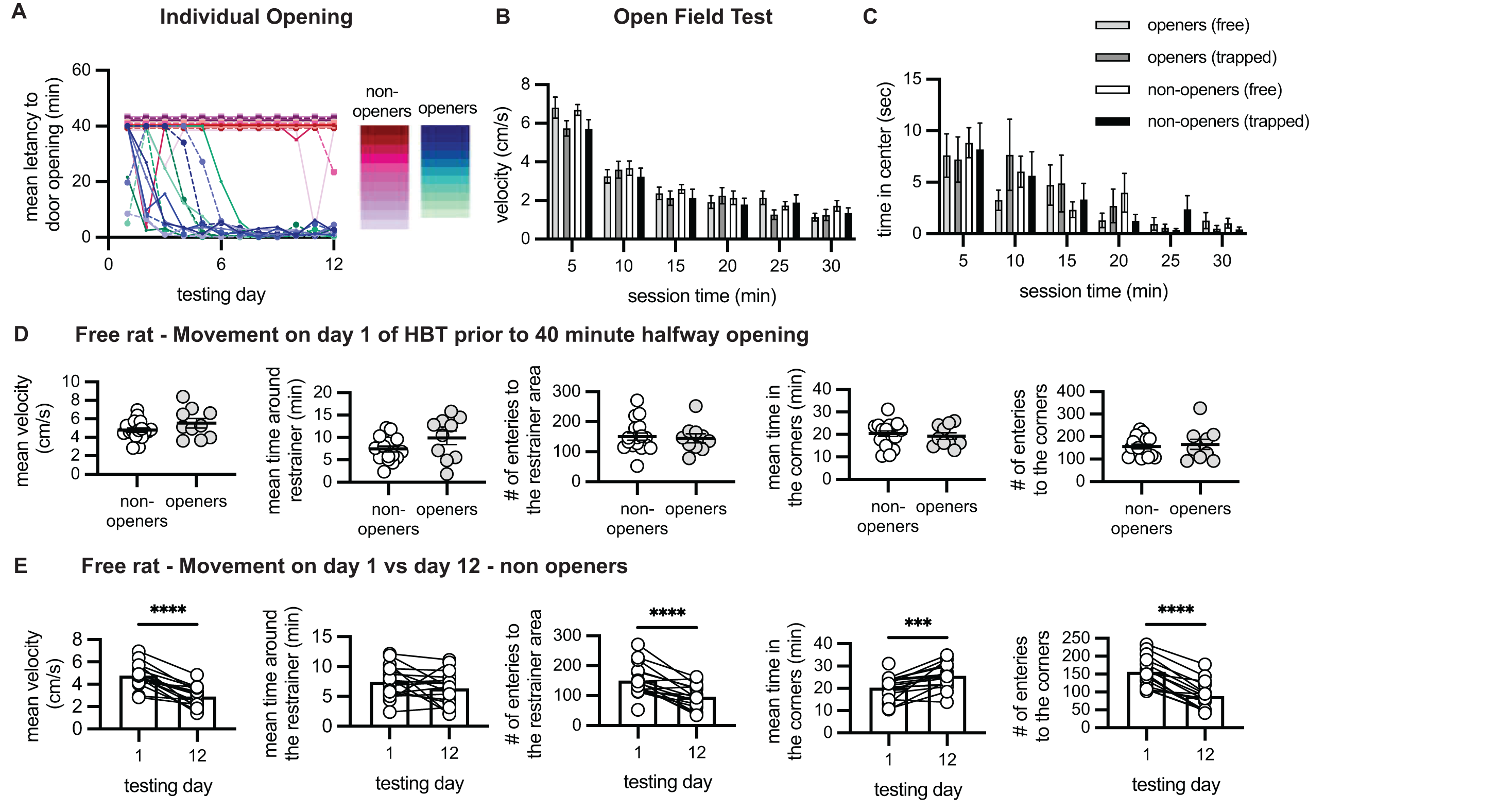

Supplement: Figure 2-1 — Detailed door-opening and movement data. A) Mean latency to door-opening across testing sessions for each individual animal. B-C) Velocity and time spent in the center of the 30-minute open field test did not differ across any condition. D) Movement patterns for the free rat on day 1 of helping (including velocity, time and number of entries to the restrainer area and time and number of entries to the corner) did not differ between nonopeners and openers. E) For non-openers, movement patterns were altered by day 12, with reduced velocity and entries into the restrainer zone, and more entries and time spent in the corners. Download Figure 2-1, TIF file. [file jneuro-45-e0845242025-s001.tif]

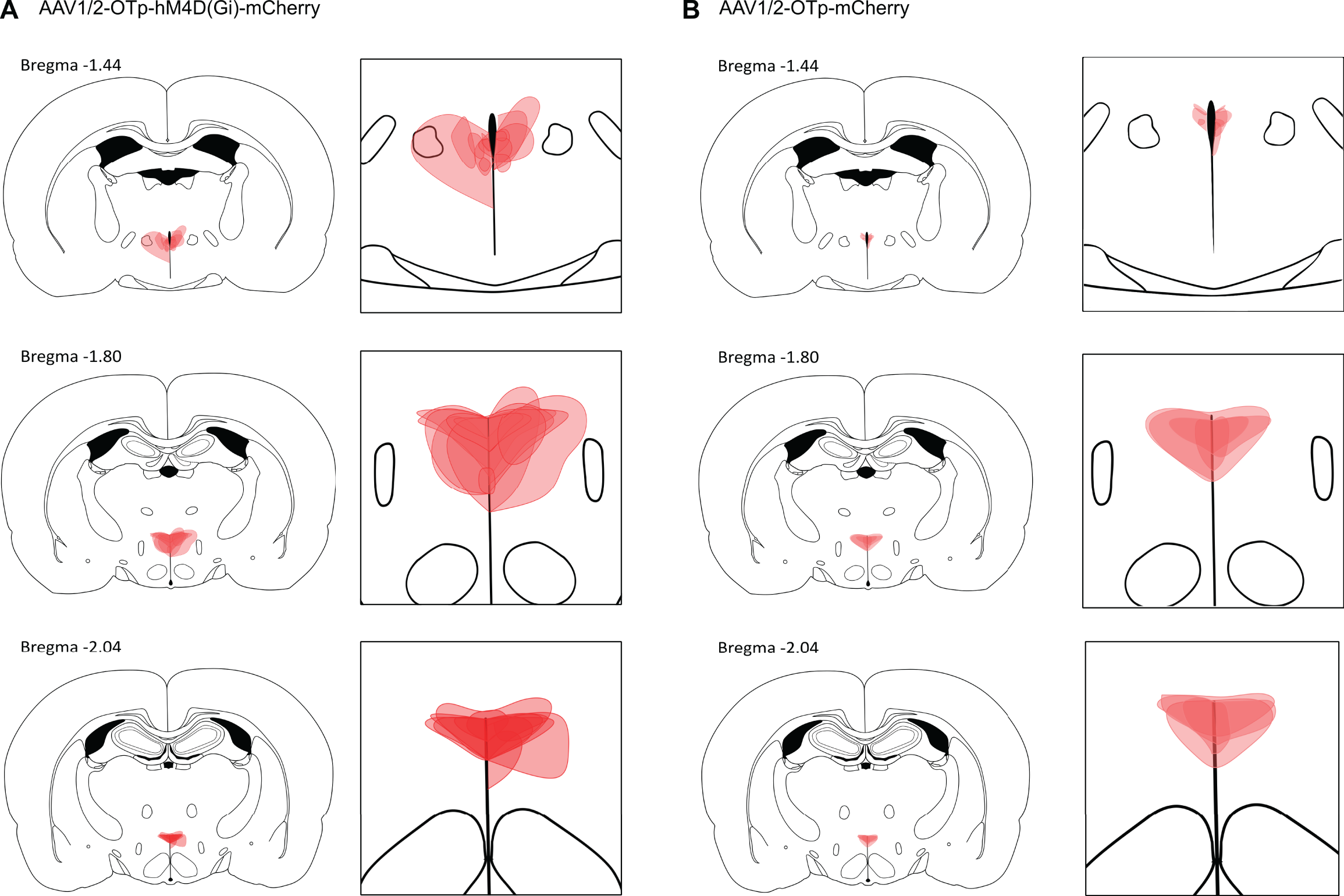

Supplement: Figure 4-1 — Injection summary. Viral injection summary in the PVN across all 12 rats for Experiment 2, including the hMRD(Gi) rats A, and the mCherry control rats B. Download Figure 4-1, TIF file. [file jneuro-45-e0845242025-s003.tif]

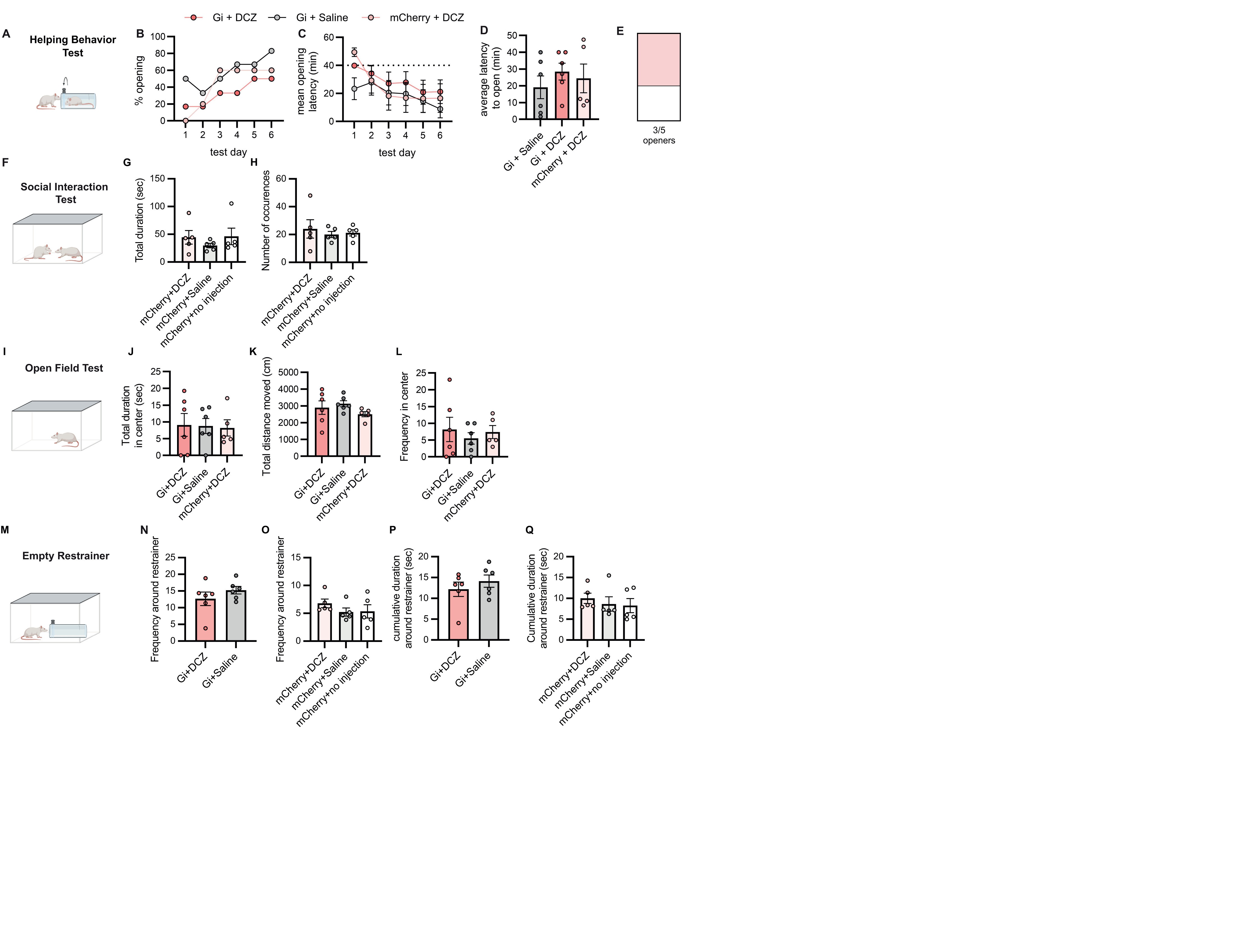

Supplement: Figure 4-2 — Control conditions for viral manipulation. A) Illustration of HBT set up. Percent-door openings increased)B) and latency to open decreased)C) across testing days for all three experimental groups. The dashed line at 40 minutes indicates when experimenters opened the door halfway. D) Average latency to open across the testing days. E) Proportion of mCherry-DCZ rats (60%, 3/5) that became openers. F) A 10-minute social interaction test was conducted after the HBT in the mCherry-expressing rats to test whether DCZ administration or injection stress impacted sociability. No differences were observed in the duration (G) or number of social interactions (H). I) An Open Field Test was conducted prior to the HBT and without any pharmacological treatment to assess for effects of viral expression on activity measures. No differences were observed across the three conditions in the total duration in center (J) total distance moved (K) and the frequency of entries into the center (L) M) A 10-minute empty restrainer test was conducted after the HBT to test for an effect of OXT inhibition on non-social activity levels. No effect was observed in frequency around the restrainer in the hM4D-Gi groups (N) or mCherry conditions (O) nor was there differences in the cumulative duration spent around the restrainer (P,Q). Download Figure 4-2, TIF file. [file jneuro-45-e0845242025-s004.tif]

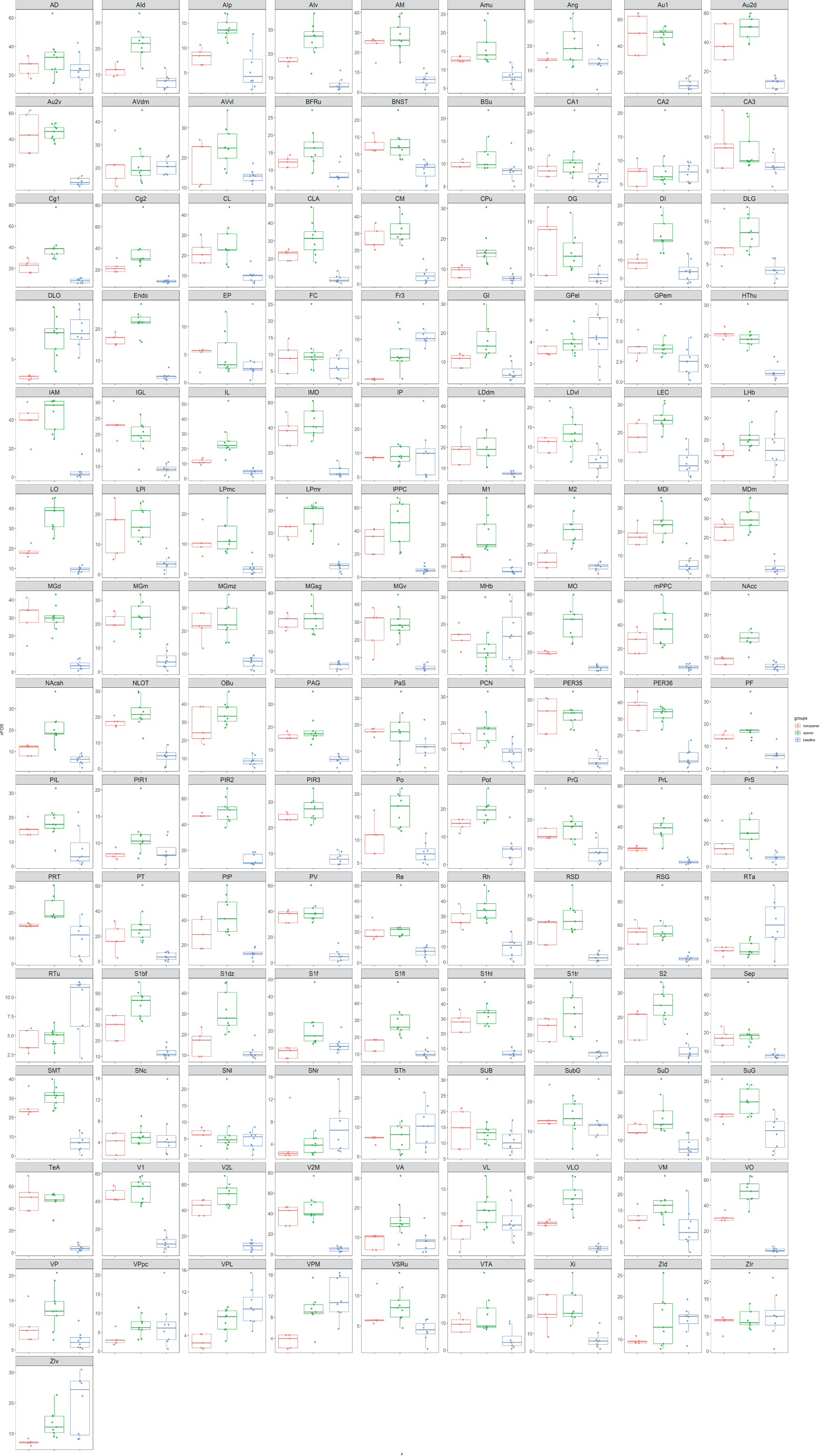

Supplement: Figure 5-1 — Visualization of c-Fos data. Box-plots showing c-Fos data for non-openers (red), compared to openers (green), and baseline animals (blue) by brain region. Download Figure 5-1, TIF file. [file jneuro-45-e0845242025-s005.tif]

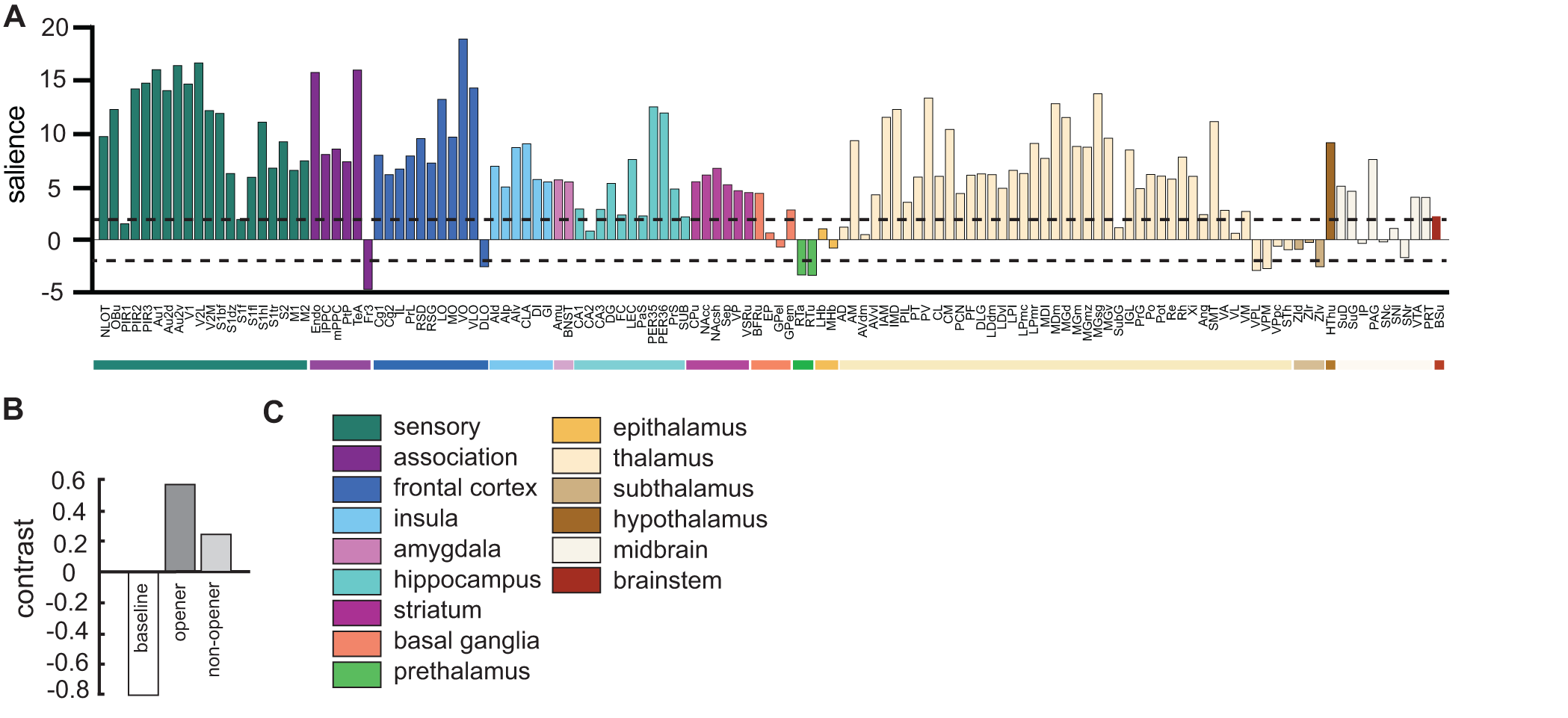

Supplement: Figure 5-2 — Brain pattern of opener and non-openers contasted with baseline. A) Partial least square (PLS) task analysis, including the baseline condition. Animals that underwent the HBT showed increased activity in nearly all brain regions compared to an untested baseline. Regions that cross the dashed lines significantly (p < 0.05) contributed to this pattern. B) PLS contrast graph: openers and non-openers showed distinct patterns of neural activity, and contrasted significantly with the brain-wide activity pattern observed in the baseline condition C) Legend of brain region categories coded by color, as seen in the main figure. Download Figure 5-2, TIF file. [file jneuro-45-e0845242025-s006.tif]

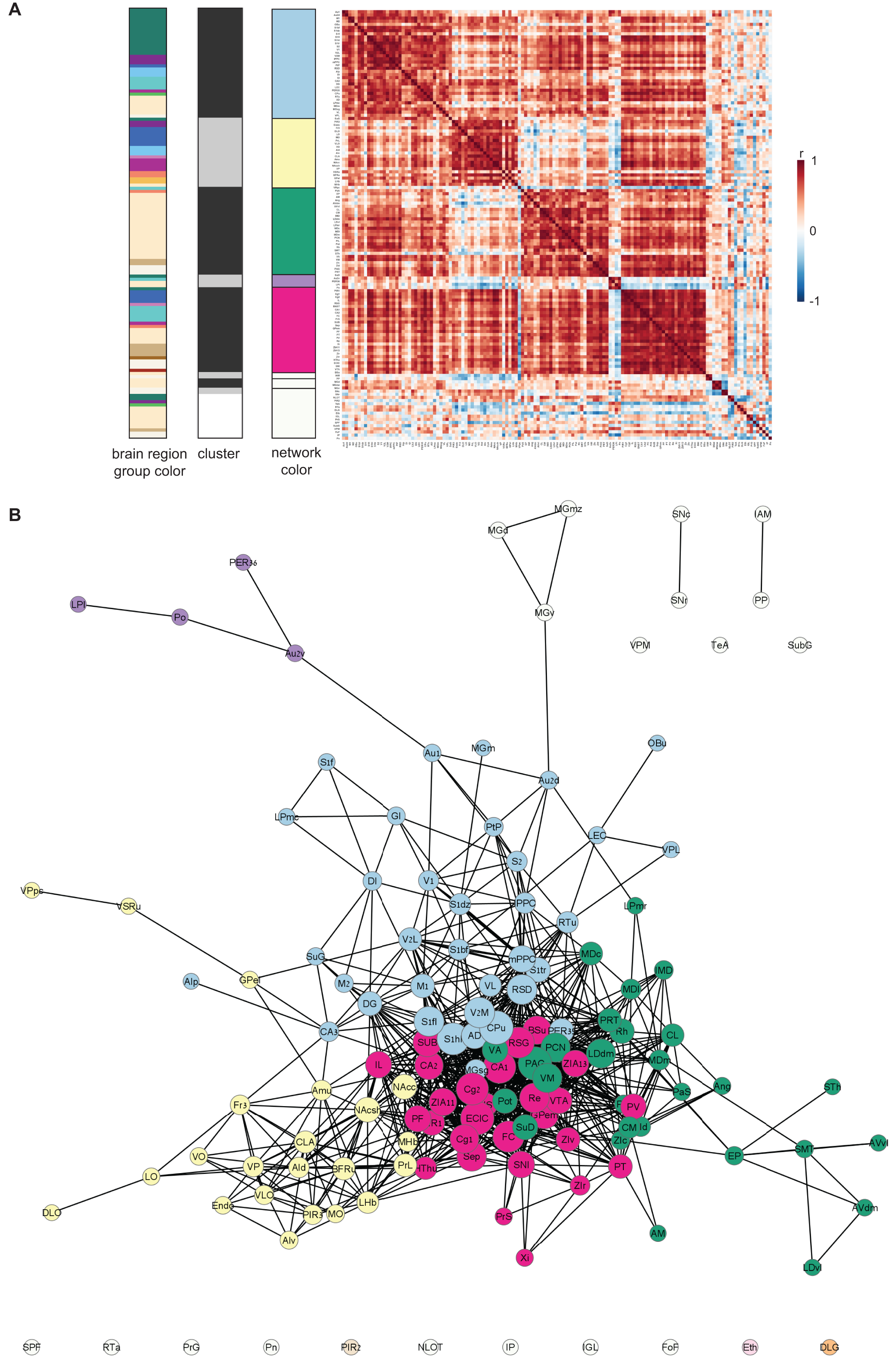

Supplement: Figure 5-3 — Network analysis of openers. In order to identify the functional connectivity involved specifically in adult helping behavior, a network analysis was conducted based on correlations between c-Fos + cells in all brain regions of helper (opener) rats. A) An inter-region Pearson’s pairwise correlation matrix. Red indicates a positive correlation, blue indicates a negative correlation. Four major clusters were identified (blue, yellow, green and pink). Coloring according to brain region group (see Figure 3I or Figure 5-2 for legend), and according to the network map shown below. B) Network graph for openers. Solid lines connecting regions denote the top 10% of positive correlations. Cluster 1 (blue) was composed mainly of sensory regions (auditory, visual, motor, & somatosensory cortices), as well as the insular cortex, some hippocampal regions (DG, CA3, perirhinal), the medial geniculate and the caudate and putamen (CPu). Cluster 2 (yellow) was composed of core regions of the prosocial response network described previously (Ben-Ami Bartal et al., 2021; Breton et al., 2022), including the NAc shell and core, OFC (VO, MO, VLO, LO, DLO), AI (Aid, AIv), PrL, claustrum, amygdala, lateral and medial habenula, ventral pallidum, olfactory regions, basal forebrain, and the frontal association area. Cluster 3 (green) included mainly thalamic regions, the periaqueductal grey (PAG) region, and regions of the zona incerta (ZI). Lastly, Cluster 4 (pink) included frontal cortex regions such as the ACC and IL, the retrosplenial cortex (RSG), some hippocampal regions (CA1, CA2, SUB), the septum, BNST, thalamic & subthalamic nuclei, hypothalamic regions, as well as regions of the midbrain (substantia nigra (SN) & ventral tegmental area (VTA)) and brainstem. This cluster was most centrally located on the network and served as a connection point between the three other clusters. Download Figure 5-3, TIF file. [file jneuro-45-e0845242025-s007.tif]
