# Supplementary material for: Neural and Behavioral Correlates of Individual Variability in Rat Helping Behavior: A Role for Social Affiliation and Oxytocin Receptors
Source: J Neurosci. 2025 Apr 28;45(22):e0845242025. doi: 10.1523/JNEUROSCI.0845-24.2025 (PMC12121707; doi:10.1523/JNEUROSCI.0845-24.2025)
Supplement: Table 3-1 — Genes of interest. List of a priori genes analyzed in Figure 3. Download Table 3-1, DOCX file. [file jneuro-45-e0845242025-s002.docx]

|  | NaC | | | AI | |
| --- | --- | --- | --- | --- | --- |
| Gene | **Log2 Fold-change** | | **P.val** | **Log2 Fold-change** | **P.val** |
| *Oxtr* | **2.60** | **0.01** | | 0.95 | 0.46 |
| *Drd1* | 0.96 | 0.34 | | 0.93 | 0.73 |
| *Drd2* | 0.96 | 0.38 | | 0.91 | 0.77 |
| *CrhR1* | 1.44 | 0.13 | | 0.92 | 0.80 |
| *Fosl1* | **2.07** | **0.02** | | 0.85 | 0.39 |
| *Nr3c1* | 0.96 | 0.12 | | **1.19** | **0.03** |

Table 3-1. List of *a priori* genes.
