# Supplementary material for: Neural and Behavioral Correlates of Individual Variability in Rat Helping Behavior: A Role for Social Affiliation and Oxytocin Receptors
Source: J Neurosci. 2025 Apr 28;45(22):e0845242025. doi: 10.1523/JNEUROSCI.0845-24.2025 (PMC12121707; doi:10.1523/JNEUROSCI.0845-24.2025)
Supplement: Table 3-2 — Altered gene expression. Top 25 statistically significantly up-regulated and down-regulated transcription factor binding motifs (TFBMs) in promoters of differentially expressed genes within the nucleus accumbens (NAc) and anterior insula (AI). Positive values indicate increased expression in openers relative to non-openers. Download Table 3-2, DOCX file. [file jneuro-45-e0845242025-s010.docx]

| **NAC** | | | | | |
| --- | --- | --- | --- | --- | --- |
| **TFBM (up)** | **Mean log ratio** | **p value** | **TFBM (down)** | **Mean log ratio** | **p value** |
| V$KROX_Q6 | 1.6558 | 0.0001 | V$ATF_B | -1.047 | 0.0015 |
| V$ZNF219_01 | 1.3623 | 0.0021 | V$ATF_01 | -1.0227 | 0.0001 |
| V$HOXC8_01 | 1.1637 | 0.0071 | V$CREB_02 | -0.9969 | 0.0052 |
| V$POU1F1_Q6 | 1.1528 | 0.0001 | V$ZIC1_01 | -0.9879 | 0.0001 |
| V$TBX5_Q5 | 1.1428 | 0.0064 | V$CREBP1_Q2 | -0.9836 | 0.0051 |
| V$CNOT3_01 | 1.0398 | 0.0068 | V$CREBATF_Q6 | -0.9534 | 0 |
| V$ATATA_B | 1.0239 | 0.0131 | V$E2F1DP1RB_01 | -0.9253 | 0.0151 |
| V$HOXD3_01 | 1.0119 | 0.018 | V$CREB_Q4 | -0.8967 | 0.0064 |
| V$IPF1_03 | 0.9405 | 0.0004 | V$CREB_01 | -0.8531 | 0.0001 |
| V$SP4_Q5 | 0.9071 | 0 | V$GC_01 | -0.8217 | 0.0064 |
| V$SP1_Q2_01 | 0.854 | 0 | V$MYC_Q2 | -0.7874 | 0.0006 |
| V$SP1SP3_Q4 | 0.8533 | 0.0007 | V$USF_01 | -0.7605 | 0.0023 |
| V$SOX9_B1 | 0.7942 | 0.0007 | V$E2F1_Q4 | -0.7597 | 0.0169 |
| V$OCT1_08 | 0.7706 | 0.135 | V$UF1H3BETA_Q6 | -0.7561 | 0.1084 |
| V$NF1_Q6_01 | 0.7665 | 0.0011 | V$HOXA7_01 | -0.7419 | 0.0001 |
| V$AP1_Q4 | 0.7551 | 0 | V$CLOCKBMAL_Q6 | -0.7369 | 0.0009 |
| V$LHX3_01 | 0.7325 | 0.0583 | V$MYCMAX_02 | -0.7239 | 0.0056 |
| V$LEF1TCF1_Q4 | 0.7318 | 0.0007 | V$HNF4_Q6 | -0.7045 | 0.0005 |
| V$PAX7_01 | 0.6917 | 0.0002 | V$FOXM1_01 | -0.7017 | 0.0001 |
| V$TATA_C | 0.649 | 0.0019 | V$MYCMAX_03 | -0.6929 | 0.0031 |
| V$CKROX_Q2 | 0.6362 | 0 | V$SP1_Q6 | -0.687 | 0.0187 |
| V$AP1FJ_Q2 | 0.6354 | 0 | V$CREB_Q4_01 | -0.6867 | 0.001 |
| V$GATA3_02 | 0.5994 | 0.0072 | V$E2F1_Q6 | -0.6842 | 0.0326 |
| V$KAISO_01 | 0.5993 | 0.0538 | V$AP3_Q6 | -0.6782 | 0 |
| **AI** | | | | | |
| **TFBM (up)** | **Mean log ratio** | **p value** | **TFBM (down)** | **Mean log ratio** | **p value** |
| V.HOXA13_02 | 0.87758961 | 0.0007 | V.HDX_01 | -1.33959863 | 0 |
| V.E2F4DP1_01 | 0.77849003 | 0.0109 | V.ICSBP_Q6 | -0.88096488 | 0 |
| V.STAT5A_01 | 0.74757366 | 0 | V.OCT_C | -0.80824998 | 0.0001 |
| V.IPF1_03 | 0.71024876 | 0.0486 | V.GADP_01 | -0.75351258 | 0 |
| V.MYCMAX_02 | 0.6993649 | 0.0004 | V.UF1H3BETA_Q6 | -0.69829853 | 0 |
| V.CHX10_01 | 0.68623627 | 0.0249 | V.TBX5_Q5 | -0.68804172 | 0 |
| V.E2F_02 | 0.58791368 | 0.0303 | V.MEF2_05 | -0.68032805 | 0.0004 |
| V.E2F1DP1RB_01 | 0.58210072 | 0.0218 | V.HMX1_01 | -0.61891849 | 0.0081 |
| V.P53_02 | 0.56891101 | 0 | V.CP2_02 | -0.61599323 | 0.0004 |
| V.STAT5B_01 | 0.53841732 | 0.0063 | V.NKX3A_01 | -0.58992233 | 0.0003 |
| V.ETS1_B | 0.52892685 | 0.0006 | V.RBPJK_01 | -0.57797465 | 0.0008 |
| V.STAT_01 | 0.5174472 | 0.0003 | V.LDSPOLYA_B | -0.57229938 | 0.0003 |
| V.NFY_Q6 | 0.51671654 | 0.0077 | V.HIF1_Q5 | -0.53736018 | 0.0005 |
| V.PROP1_01 | 0.51056809 | 0.0228 | V.PAX_Q6 | -0.53276098 | 0.0068 |
| V.NFY_01 | 0.50975479 | 0.0017 | V.GR_Q6 | -0.51029954 | 0 |
| V.HNF6_Q6 | 0.49147408 | 0.0324 | V.MEF2_Q6_01 | -0.49734942 | 0.0137 |
| V.CAAT_01 | 0.4839906 | 0.0027 | V.BRN2_01 | -0.47988718 | 0 |
| V.TITF1_Q3 | 0.45442331 | 0.0523 | V.T3R_01 | -0.45543744 | 0.083 |
| V.CETS1P54_03 | 0.45141393 | 0.0126 | V.ETS2_B | -0.45183229 | 0.0003 |
| V.AML_Q6 | 0.44855058 | 0.0106 | V.ARNT_02 | -0.44485623 | 0.0291 |
| V.E2F1DP1_01 | 0.43528292 | 0.0473 | V.VMYB_01 | -0.43467575 | 0 |
| V.NRF1_Q6 | 0.42990587 | 0.0469 | V.MYOD_Q6_01 | -0.4124555 | 0.0026 |
| V.GATA3_03 | 0.4276832 | 0.0089 | V.NANOG_01 | -0.41002324 | 0.0155 |
| V.NFY_Q6_01 | 0.37870407 | 0.0088 | V.MYOD_01 | -0.40738891 | 0.0033 |
| V.HEB_Q6 | 0.36202429 | 0 | V.MAF_Q6_01 | -0.3975848 | 0.0413 |

Table 3-2. Top 25 statistically significantly up-regulated and down-regulated transcription factor binding motifs (TFBMs) in promoters of differentially expressed genes within the nucleus accumbens (NAc) and anterior insula (AI). Positive values indicate increased expression in openers relative to non-openers.
