# Supplementary material for: Neural and Behavioral Correlates of Individual Variability in Rat Helping Behavior: A Role for Social Affiliation and Oxytocin Receptors
Source: J Neurosci. 2025 Apr 28;45(22):e0845242025. doi: 10.1523/JNEUROSCI.0845-24.2025 (PMC12121707; doi:10.1523/JNEUROSCI.0845-24.2025)
Supplement: Table 5-1 — Brain region list. Detailed list of 137 brain regions analyzed and used in Figures 4-5. Brain region abbreviations and full names, organized by presentation and brain region category. Download Table 5-1, DOCX file. [file jneuro-45-e0845242025-s008.docx]

| Acronym | Name | Region # | Category |
| --- | --- | --- | --- |
| NLOT | Nucleus of the lateral olfactory tract | 1 | Sensory |
| OBu | Olfactory bulb, unspecified | 2 | Sensory |
| PIR1 | Piriform cortex, layer 1 | 3 | Sensory |
| PIR2 | Piriform cortex, layer 2 | 4 | Sensory |
| PIR3 | Piriform cortex, layer 3 | 5 | Sensory |
| Au1 | Primary auditory area | 6 | Sensory |
| Au2d | Secondary auditory area, dorsal part | 7 | Sensory |
| Au2v | Secondary auditory area, ventral part | 8 | Sensory |
| V1 | Primary visual area | 9 | Sensory |
| V2L | Secondary visual area, lateral part | 10 | Sensory |
| V2M | Secondary visual area, medial part | 11 | Sensory |
| S1bf | Primary somatosensory area, barrel field | 12 | Sensory |
| S1dz | Primary somatosensory area, dysgranular zone | 13 | Sensory |
| S1f | Primary somatosensory area, face representation | 14 | Sensory |
| S1fl | Primary somatosensory area, forelimb representation | 15 | Sensory |
| S1hl | Primary somatosensory area, hindlimb representation | 16 | Sensory |
| S1tr | Primary somatosensory area, trunk representation | 17 | Sensory |
| S2 | Secondary somatosensory area | 18 | Sensory |
| M1 | Primary motor area | 19 | Sensory |
| M2 | Secondary motor area | 20 | Sensory |
| Endo | Endopiriform nucleus | 21 | Association |
| lPPC | Parietal association cortex, lateral area | 22 | Association |
| mPPC | Parietal association cortex, medial area | 23 | Association |
| PtP | Parietal association cortex, posterior area | 24 | Association |
| TeA | Temporal association cortex | 25 | Association |
| Fr3 | Frontal association area 3 | 26 | Association |
| Cg1 | Cingulate area 1 | 27 | Frontal cortex |
| Cg2 | Cingulate area 2 | 28 | Frontal cortex |
| IL | Infralimbic area | 29 | Frontal cortex |
| PrL | Prelimbic area | 30 | Frontal cortex |
| RSD | Retrosplenial dysgranular area | 31 | Frontal cortex |
| RSG | Retrosplenial granular area | 32 | Frontal cortex |
| LO | Lateral orbital area | 33 | Frontal cortex |
| MO | Medial orbital area | 34 | Frontal cortex |
| VO | Ventral orbital area | 35 | Frontal cortex |
| VLO | Ventrolateral orbital area | 36 | Frontal cortex |
| DLO | Dorsolateral orbital area | 37 | Frontal cortex |
| AId | Agranular insular cortex dorsal area | 38 | Insula |
| AIp | Agranular insular cortex, posterior area | 39 | Insula |
| AIv | Agranular insular cortex, ventral area | 40 | Insula |
| CLA | Claustrum | 41 | Insula |
| DI | Dysgranular insular cortex | 42 | Insula |
| GI | Granular insular cortex | 43 | Insula |
| Amu | Amygdaloid area, unspecified | 44 | Amygdala |
| BNST | Bed nucleus of the stria terminalis | 45 | Amygdala |
| CA1 | Cornu ammonis 1 | 46 | Hippocampal formation |
| CA2 | Cornu ammonis 2 | 47 | Hippocampal formation |
| CA3 | Cornu ammonis 3 | 48 | Hippocampal formation |
| DG | Dentate gyrus | 49 | Hippocampal formation |
| FC | Fasciola cinereum | 50 | Hippocampal formation |
| LEC | Lateral entorhinal cortex | 51 | Hippocampal formation |
| PaS | Parasubiculum | 52 | Hippocampal formation |
| PER35 | Perirhinal area 35 | 53 | Hippocampal formation |
| PER36 | Perirhinal area 36 | 54 | Hippocampal formation |
| PrS | Presubiculum | 55 | Hippocampal formation |
| SUB | Subiculum | 56 | Hippocampal formation |
| CPu | Caudate putamen | 57 | Striatum |
| NAcc | Nucleus accumbens, core | 58 | Striatum |
| NAcsh | Nucleus accumbens, shell | 59 | Striatum |
| Sep | Septal region | 60 | Striatum |
| VP | Ventral pallidum | 61 | Striatum |
| VSRu | Ventral striatal region, unspecified | 62 | Striatum |
| BFRu | Basal forebrain region, unspecified | 63 | Basal Ganglia |
| EP | Entopeduncular nucleus | 64 | Basal Ganglia |
| GPel | Globus pallidus external, lateral part | 65 | Basal Ganglia |
| GPem | Globus pallidus external, medial part | 66 | Basal Ganglia |
| RTa | Reticular (pre)thalamic nucleus, auditory segment | 67 | Prethalamus |
| RTu | Reticular (pre)thalamic nucleus, unspecified | 68 | Prethalamus |
| LHb | Lateral habenular nucleus | 69 | Epithalamus |
| MHb | Medial habenular nucleus | 70 | Epithalamus |
| AD | Anterodorsal thalamic nucleus | 71 | Thalamus |
| AM | Anteromedial thalamic nucleus | 72 | Thalamus |
| AVdm | Anteroventral thalamic nucleus, dorsomedial part | 73 | Thalamus |
| AVvl | Anteroventral thalamic nucleus, ventrolateral part | 74 | Thalamus |
| IAM | Interanteromedial thalamic nucleus | 75 | Thalamus |
| IMD | Intermediodorsal thalamic nucleus | 76 | Thalamus |
| PIL | Posterior intralaminar nucleus | 77 | Thalamus |
| PT | Parataenial thalamic nucleus | 78 | Thalamus |
| PV | Paraventricular thalamic nuclei (anterior and posterior) | 79 | Thalamus |
| SPF | Subparafascicular nucleus | 80 | Thalamus |
| CL | Central lateral thalamic nucleus | 81 | Thalamus |
| CM | Central medial thalamic nucleus | 82 | Thalamus |
| Eth | Ethmoid-Limitans nucleus | 83 | Thalamus |
| PCN | Paracentral thalamic nucleus | 84 | Thalamus |
| PF | Parafascicular thalamic nucleus | 85 | Thalamus |
| DLG | Dorsal lateral geniculate nucleus | 86 | Thalamus |
| LDdm | Laterodorsal thalamic nucleus, dorsomedial part | 87 | Thalamus |
| LDvl | Laterodorsal thalamic nucleus, ventrolateral part | 88 | Thalamus |
| LPl | Lateral posterior thalamic nucleus, lateral part | 89 | Thalamus |
| LPmc | Lateral posterior thalamic nucleus, mediocaudal part | 90 | Thalamus |
| LPmr | Lateral posterior thalamic nucleus, mediorostral part | 91 | Thalamus |
| MDc | Mediodorsal thalamic nucleus, central part | 92 | Thalamus |
| MDl | Mediodorsal thalamic nucleus, lateral part | 93 | Thalamus |
| MDm | Mediodorsal thalamic nucleus, medial part | 94 | Thalamus |
| MGd | Medial geniculate body, dorsal division | 95 | Thalamus |
| MGm | Medial geniculate body, medial division | 96 | Thalamus |
| MGmz | Medial geniculate body, marginal zone | 97 | Thalamus |
| MGsg | Medial geniculate body, suprageniculate nucleus | 98 | Thalamus |
| MGv | Medial geniculate body, ventral division | 99 | Thalamus |
| SubG | Subgeniculate nucleus | 100 | Thalamus |
| IGL | Intergeniculate leaflet | 101 | Thalamus |
| PrG | Pregeniculate nucleus | 102 | Thalamus |
| Po | Posterior thalamic nucleus | 103 | Thalamus |
| Pot | Posterior thalamic nuclear group, triangular part | 104 | Thalamus |
| Re | Reuniens thalamic nucleus | 105 | Thalamus |
| Rh | Rhomboid thalamic nucleus | 106 | Thalamus |
| Xi | Xiphoid thalamic nucleus | 107 | Thalamus |
| Ang | Angular thalamic nucleus | 108 | Thalamus |
| SMT | Submedius thalamic nucleus | 109 | Thalamus |
| VA | Ventral anterior thalamic nucleus | 110 | Thalamus |
| VL | Ventrolateral thalamic nucleus | 111 | Thalamus |
| VM | Ventromedial thalamic nucleus | 112 | Thalamus |
| VPL | Ventral posterolateral thalamic nucleus | 113 | Thalamus |
| VPM | Ventral posteromedial thalamic nucleus | 114 | Thalamus |
| VPpc | Ventral posterior nucleus of the thalamus, parvicellular part | 115 | Thalamus |
| STh | Subthalamic nucleus | 116 | Thalamus |
| ZIA11 | Zona incerta, A11 dopamine cells | 117 | Subthalamus |
| ZIA13 | Zona incerta, A13 dopamine cells | 118 | Subthalamus |
| ZIc | Zona incerta, caudal part | 119 | Subthalamus |
| ZId | Zona incerta, dorsal part | 120 | Subthalamus |
| ZIr | Zona incerta, rostral part | 121 | Subthalamus |
| ZIv | Zona incerta, ventral part | 122 | Subthalamus |
| FoF | Fields of Forel | 123 | Subthalamus |
| HThu | Hypothalamic region, unspecified | 124 | Hypothalamus |
| ECIC | Inferior colliculus, external cortex | 125 | Midbrain |
| SuD | Deeper layers of the superior colliculus | 126 | Midbrain |
| SuG | Superficial gray layer of the superior colliculus | 127 | Midbrain |
| IP | Interpeduncular nucleus | 128 | Midbrain |
| PAG | Periaqueductal gray | 129 | Midbrain |
| PP | Peripeduncular nucleus | 130 | Midbrain |
| SNc | Substantia nigra, compact part | 131 | Midbrain |
| SNl | Substantia nigra, lateral part | 132 | Midbrain |
| SNr | Substantia nigra, reticular part | 133 | Midbrain |
| VTA | Ventral tegmental area | 134 | Midbrain |
| Pn | Pontine nuclei | 135 | Midbrain |
| PRT | Pretectal region | 136 | Midbrain |
| BSu | Brainstem, unspecified | 137 | Brainstem |

**Table 5-1.** Brain region abbreviations and full names, organized by presentation and brain region category.
