# Supplementary material for: Neural and Behavioral Correlates of Individual Variability in Rat Helping Behavior: A Role for Social Affiliation and Oxytocin Receptors
Source: J Neurosci. 2025 Apr 28;45(22):e0845242025. doi: 10.1523/JNEUROSCI.0845-24.2025 (PMC12121707; doi:10.1523/JNEUROSCI.0845-24.2025)
Supplement: Table 6-1 — Correlations of ROIs with social behavior. The 36 brain regions that significantly contributed to the contrast between openers and non-openers were tested for correlations with social interaction on the first day of the HBT. Brain region category, region abbreviation and Pearson’s correlations are reported here, with statistically significant correlations in bold. Download Table 6-1, DOCX file. [file jneuro-45-e0845242025-s009.docx]

| **Category** | **Brain region** | **Correlation with social interaction duration – First HBT** |
| --- | --- | --- |
| Frontal cortex | Cg1 | r=0.5441, p=0.0546 |
|  | PrL | r=0.3628, p=0.2231 |
|  | IL | r=0.4149, p=0.1587 |
|  | MO | r=0.3897, p=0.1881 |
|  | LO | r=0.5269, p=0.0643 |
|  | VO | r=0.4515, p=0.1216 |
|  | DLO | r=0.3970, p=0.1792 |
|  | VLO | r=0.5127, p=0.0732 |
| Sensory | M1 | **r=0.7422, p=0.0037** |
|  | M2 | **r=0.6299, p=0.0210** |
|  | S1bf | **r=0.6013, p=0.0297** |
|  | S1fl | **r=0.6931, p=0.0086** |
|  | S1dz | **r=0.6799, p=0.0106** |
|  | S1f | **r=0.6569, p=0.0147** |
| Insula | DI | **r=0.7397, p=0.0039** |
|  | GI | **r=0.7781, p=0.0017** |
|  | Aid | r=0.5015 p=0.0808 |
|  | AIv | r=0.2408, p=0.428 |
|  | AIp | **r=0.6329 p=0.0011** |
| Association | Fr3 | **r=0.5933, p=0.0326** |
|  | Endo | r=0.1227, p=0.6896 |
| Epithalamus | LHb | **r=0.5604, p=0.0464** |
| Striatum | CPu | r=0.5253, p=0.0652 |
|  | NAcSh | r=0.5118, p=0.0738 |
|  | NAcC | r=0.4890, p=0.0899 |
| Thalamus | VA | **r=0.5886, p=0.0343** |
|  | VPpc | r=0.4890, p=0.0899 |
|  | Pot | r=0.5231, p=0.0666 |
|  | PO | r=0.3006, p=0.3183 |
|  | Eth | r=0.2529, p=0.4045 |
|  | VL | r=0.4464, p=0.1262 |
|  | VPM | r=0.1290, p=0.6744 |
|  | VPL | r=0.2768, p=0.3598 |
| Subthalamus | FoF | r=0.1524, p=0.6192 |
|  | Ziv | r=0.2659, p=0.3799 |
| Midbrain | PRT | r=0.3198, p=0.2868 |

**Table 6-1.** The 36 brain regions that significantly contributed to the contrast between openers and non-openers were tested for correlations with social interaction on the first day of the HBT. Brain region category, region abbreviation and Pearson’s correlations are reported here, with statistically significant correlations in bold.
